# Effect of remediation reagents on bacterial composition and ecological function in black-odorous water sediments

**DOI:** 10.1007/s00203-022-02871-4

**Published:** 2022-04-24

**Authors:** Dong Xia, Hanbin Zhao, Sohei Kobayashi, Qi Mi, Aimin Hao, Yasushi Iseri

**Affiliations:** 1 College of Life and Environmental Science, Wenzhou University, Wenzhou, 325035 Zhejiang China; 2 National and Local Joint Engineering Research Center of Ecological Treatment Technology for Urban Water Pollution, Wenzhou University, Wenzhou, 325035 Zhejiang China; 3 Nanjing Guoxing Environmental Protection Industry Research Institute Co. LTD, Nanjing, 211899 China

**Keywords:** Remediation reagent, Bacterial community, Functional group, In situ, Black-odorous sediment

## Abstract

Black-odorous urban water bodies and sediments pose a serious environmental problem. In this study, we conducted microcosm batch experiments to investigate the effect of remediation reagents (magnesium hydroxide and calcium nitrate) on native bacterial communities and their ecological functions in the black-odorous sediment of urban water. The dominant phyla (Proteobacteria, Actinobacteria, Chloroflexi, and Planctomycetes) and classes (Alphaproteobacteria, Betaproteobacteria, and Gammaproteobacteria, Actinobacteria, Anaerolineae, and Planctomycetia) were determined under calcium nitrate and magnesium hydroxide treatments. Functional groups related to aerobic metabolism, including aerobic chemoheterotrophy, dark sulfide oxidation, and correlated dominant genera (*Thiobacillus*, *Lysobacter*, Gp16, and *Gaiella*) became more abundant under calcium nitrate treatment, whereas functional genes potentially involved in dissimilatory sulfate reduction became less abundant. The relative abundance of chloroplasts, fermentation, and correlated genera (*Desulfomonile* and unclassified Cyanobacteria) decreased under magnesium hydroxide treatment. Overall, these results indicated that calcium nitrate addition improved hypoxia-related reducing conditions in the sediment and promoted aerobic chemoheterotrophy.

**Supplementary Information:**

The online version contains supplementary material available at 10.1007/s00203-022-02871-4.

## Introduction

With the rapid development and modernization of industry and agriculture, the number of water bodies affected by urban environmental pollution has been progressively increasing; this includes urban rivers, ponds, and lakes, which can be contaminated by domestic wastewater effluent, agricultural runoff, and industrial wastewater. Moreover, the continuous deterioration of water quality, characterized by turbid and odorous water, has resulted in serious damage to aquatic ecosystems in these urban water bodies, and is viewed as one of the greatest threats to urban ecosystems (Zhang et al. 2016; Zhu et al. 2016; Liu et al. 2017; Chen et al. 2022).

Hypoxia, which is caused by the accumulation of large amounts of organic pollutants in sediment and the excessive use of dissolved oxygen (DO) during decay processes, is the main factor responsible for the development of black and odorous water and sediment. The redox potential (oxidation/reduction potential, ORP) is positively correlated with DO; thus, a low ORP value can easily lead to further sediment-reducing conditions, which in turn is conducive to the accelerated growth of anaerobic bacteria (such as sulfate-reducing bacteria, SRB) and the bacterial reduction of sulfate (SO_4_^2−^) in sediments (JØrgensen 2019; Zhang et al. 2021a, b). H_2_S and other odorous gaseous sulfuric compounds produced by bacterial anaerobic respiration are the main causes of the odorous phenomenon observed in urban rivers (Liu et al. 2017; Zhao et al. 2019). Furthermore, the sediment-reducing conditions caused by a low DO and ORP can promote Fe/Mn reduction and phosphorus (P) release, where *P* can be adsorbed onto Fe and Mn oxide-hydroxide particles (Fink et al. 2016a, b, c; Gasparatos et al. 2019; Franks et al. 2021). Meanwhile, Fe(II) can also react with H_2_S to form black and low-solubility iron sulfide (FeS), which causes the sediment and overlying water to turn black in color (Liu et al. 2017).

In situ remediation is a powerful technology for treating contaminated sediments by efficiently degrading organic matter and controlling the release of pollutants to the overlying water body (Cui et al. 2018; Li et al. 2019). In addition, calcium nitrate, sodium percarbonate, and domesticated bacteria are effective reagents in remediation technologies (de Alencar et al. 2017; Pan et al. 2018; Tang et al. 2018; Shen et al. 2019). Calcium nitrate [Ca(NO_3_)_2_] is a common oxidant that has been widely used in the in situ remediation of black and malodorous sediments. Ca(NO_3_)_2_ promotes restoration through the following three mechanisms: improving the oxidation conditions of sediments, providing electron acceptors for microorganisms (such as denitrification bacteria) to accelerate their organic matter degradation activity, and controlling the diffusion of nitrogen and phosphorus nutrients to the overlying water body (Liu et al. 2017; Tang et al. 2018). Magnesium hydroxide (Mg(OH)_2_), which can turn the sediment environment weakly alkaline and affect the metabolic activity of SRB and other microorganisms related to black-odorous water, as well as the production of black-malodorous substances, has been proposed as a new environmentally friendly remediation reagent for sediment purification in marine cage culture in Japan (Nishino and Kawauchi 2003). Furthermore, Mg(OH)_2_ is also an effective antiseptic substance used to prevent corrosion and H_2_S emissions in the sewage collection pipelines of various municipalities (US Environmental Protection Agency 1974; Nielsen et al. 1998; Li et al. 2020a, b; Guan et al. 2021; Rabinovich et al. 2021). Moreover, the addition of Mg(OH)_2_ to sewage sediment can deactivate the sulfate-reducing capabilities of SRB under high pH conditions (Yoshida and Nishino 2005; Faridah et al. 2011). Therefore, Ca(NO_3_)_2_ and Mg(OH)_2_ both show potential for sediment remediation; however, previous studies related to sediment remediation have focused mainly on the effect of remediation reagents on the physicochemical properties of sediment and the overlying water, with few analyzing the differences in bacterial community structure and ecological function before and after treatment, as well as the interaction among remediation reagents, bacterial community, and ecological function (Andrade et al. 2017; Wei et al. 2017; Yang et al. 2018).

In this study, both Ca(NO_3_)_2_ and Mg(OH)_2_ were added to contaminated sediments as remediation reagents. High-throughput sequencing technology was applied to analyze the bacterial communities in the sediment and clarify the transformation of functional microbes during the remediation process. Changes in the sediment conditions, including DO, ORP, pH, total organic carbon (TOC), and acid volatile sulfide (AVS), were also examined. The goal of this study was to explore the effects of Mg(OH)_2_ and Ca(NO_3_)_2_ on bacterial diversity and community composition, as well as bacterial ecological function in sediment. The role of different functional microbes in the remediation process of sediments is also discussed.

## Materials and methods

### Experimental procedure

Sediments and overlying water were collected from one site of a pond located on the east side of an urban park, which is on the south of the Ou River that flows east near central Wenzhou City, Zhejiang Province, China. The pond is a slender shape with length and width of 300 m and 20 m, respectively, in the north–south direction, and the depth varies from 1.2 m to 2.0 m from north to south. Because there is no apparent inflow and outflow, the oxygen deficit, unpleasant odor, and black color occur often in the water body. Sediments were homogenized and stored in the dark at 4 °C for subsequent use in experiments. A total of 36 microcosms of the sediment-overlying water body were built using 1,000 mL beakers as the experimental vials. Each beaker contained 200 mL of the original sediment and 800 mL of the original overlying water. The characteristics of the original sediment were as follows: pH 7.23, ORP − 163.4 mV, DO 0.19 mg/L, electrical conductivity (EC) 686 µS/cm, water content 81.30%, AVS 3,437.0 mg/kg, and TOC 73.7 mg/g. The characteristics of the original overlying water were as follows: pH 7.50, ORP 39.80 mV, DO 0.65 mg/L, and EC 405 µS/cm. Then, 50 mL of Mg(OH)_2_ or Ca(NO_3_)_2_ suspension (with an optimal concentration value of 500 g/m^2^ and 45.3 g-N/m^2^ respectively, based on previous field experiments (Xia et al. 2018a, b; Sun et al. 2019)) was scatter injected to a depth of 2 cm below the surface of the sediment in each microcosm, except in the control microcosms. Finally, each beaker was sealed with Parafilm and stored in the dark at 25 °C. All treatments (control, Mg(OH)_2_, and Ca(NO_3_)_2_) were performed in triplicate (nine samples in total), and samples were collected on days 7, 14, 21, and 30 of the experiment. The original sediment and overlying water were used as the original samples on day 0 for all treatments.

### Determination of sediment properties

After the physicochemical measurements were recorded, the overlying water was collected using a siphoning method. Sediment measurements were then taken; the sediment was mixed well and then collected. The water and sediment samples were stored at − 20 °C until further analysis. The values of pH, ORP, DO, and EC in the sediments and overlying water were determined using a HACH HQ40d dual-input multi-parameter meter (HACH, Loveland, USA). The weight of the sediment was measured before and after drying at 105 °C for 24 h, and the water content was calculated according to the weight loss. AVS and TOC concentrations in the sediments were analyzed using the National environmental protection standard of the People’s Republic of China: Soil and Sediment-Determination of Sulfide-Methylene Blue Spectrophotometric Method (HJ833–2017) and Soil-Determination of Organic Carbon–Potassium Dichromate Oxidation Spectrophotometric Method (HJ615–2011), respectively.

### DNA extraction

DNA extraction and polymerase chain reaction (PCR) amplification were conducted as previously described (Xia et al. 2018a, b). The TIANamp Soil DNA Kit (Tiangen Biotech (Co., Ltd., Beijing, China) was used to extract microbial DNA from the sediment samples according to the manufacturer’s instructions. Extracted DNA was purified using the SanPrep DNA Gel Extraction Kit (Sangon Biotech, Shanghai, China) and a micro-spectrophotometer (SMA4000, Merinton (Beijing) Instrument, Ltd., Beijing, China) was used to determine the DNA quality and quantity.

### High-throughput sequencing of 16S rRNA and functional genes

The sulfate-reducing gene *dsrB*, which encodes the dissimilatory sulfite reductase (Dsr) in the dissimilatory sulfate reduction process, and the nitrite-reducing gene *nirS*, which encodes nitrite reductase (Nir) in the denitrification process, were used to detect the potential functional bacteria SRB and nitrate-reducing bacteria (NRB). The target regions of the *dsrB* gene (350 bp) and *nirS* gene (425 bp) were amplified using the barcoded primer pairs DSRp2060F (5ʹ- CAA CAT CGT YCA YAC CCA GGG-3ʹ) and DSR4R (5ʹ- GTG TAG CAG TTA CCG CA-3ʹ) (Geets et al. 2006) and nirScd3aF (5ʹ- GTS AAC GTS AAG GAR ACS GG-3ʹ) and nirSR3cd (5ʹ- GAS TTC GGR TGS GTC TTG A-3ʹ) (Throbäck et al. 2004). The V3-V4 hyper variable regions of the 16S rRNA genes were amplified using the barcoded primers 341F (5ʹ-CCT ACG GGN GGC WGC AG-3ʹ) and 805R (5ʹ-GAC TAC HVG GGT ATC TAA TCC-3ʹ) (Vasileiadis et al. 2012).

Double amplification was conducted to obtain high-quality PCR products. The DNA template (10–20 ng) of each sample was amplified in a 30 µL reaction system (2X Hieff^®^ Robust PCR Master Mix) in triplicate. The first amplification was performed under the following conditions: initial denaturation at 94 °C for 3 min, followed by 5 cycles of denaturation at 94 °C for 30 s, annealing at 45 °C for 20 s, and elongation at 65 °C for 30 s, and then 20 cycles of denaturation at 94 °C for 20 s, annealing at 55 °C for 20 s, and elongation at 72 °C for 30 s, with a final extension at 72 °C for 5 min. The PCR products were used as the template to initiate the second amplification under the following conditions: initial denaturation at 95 °C for 3 min, followed by 20 cycles of denaturation at 94 °C for 20 s, annealing at 55 °C for 20 s, elongation at 72 °C for 30 s, and a final extension at 72 °C for 5 min. The negative control, using ddH_2_O as the DNA template, was also amplified under the same conditions in the PCR amplification stage. Then, the PCR products were mixed with 1 × loading buffer (containing SYBR Green dye) and detected by electrophoresis on a 1.5% agarose gel. The PCR amplicons for each sample were pooled and purified using the SanPrep Column DNA Gel Extraction Kit (SK8131, Sangon Biotech, Shanghai, China). Quality was assessed on a Qubit3.0 Fluorometer (Thermo Scientific).

The Illumina MiSeq platform (Illumina MiSeq System, Illumina, Inc., California, USA) was used for sequencing, whereby 300 bp paired-end reads were generated. Paired-end reads were merged using PEAR (v0.9.8) to obtain raw tags (Zhang et al. 2014). Quality control was performed using PRINSEQ (v0.20.4) (Schmieder and Edwards 2011), which included the removal of low-quality reads and chimeric sequences and checking the bases falling out of the V3-V4 regions. High-throughput sequencing was performed at Sangon Biotech (Shanghai) Co., Ltd. (Shanghai, China).

### Processing of sequencing data

Subsequent analyses were performed in QIIME (v1.9.0) with the following modification. Based on a 97% similarity threshold, high-quality sequences were clustered to operational taxonomic units (OTUs) against the ribosomal database project (RDP) classifier database (v2.12) (Wang et al. 2007). This included a built-in chimera check performed by Usearch (v11.0.667). Alpha diversity analysis, including the ACE (Abundance-based Coverage Estimator), Chao1, Shannon, Simpson, and Coverage indices, were calculated using QIIME (v1.9.0). ACE and Chao1 indices reflect the bacterial richness, whereas Shannon and Simpson indices reflect the bacterial diversity (a higher Shannon index or lower Simpson index indicates a lower proportion of dominant bacteria abundance in the total biomass), and the coverage index represents the sequencing depth. Taxonomic information was assigned to each OTU via the native Bayesian assignment method, and the OTU numbers were normalized to equal reads per sample for subsequent analysis. Hierarchical clustering based on Ward's method and a comparison of the group distance based on weighted UniFrac similarity was performed using the vegan package in R (v.3.5.3). The various functional contributions of the bacteria in the sediment of different treatment microcosms were explored based on OTUs using Functional Annotation of Prokaryotic Taxa (FAPROTAX) software (v1.2.1) (Louca et al. 2016) and Phylogenetic Investigation of Communities by Reconstruction of Unobserved States (PISRUSt) software (v1.1.4) (Langille et al. 2013).

### Statistical analysis

Welch’s two-sample *t* test was performed to evaluate the differences in sediment variables (DO, pH, ORP, AVS, TOC, total bacteria, SRB, and NRB) and the abundance of FAPROTAX and PICRUSt function prediction among the control, Mg(OH)_2_, and Ca(NO_3_)_2_ treatments. Non-parametric multivariate analysis of variance (ADONIS) was performed using the “vegan” R package, and 999 displacement tests were performed to determine whether the differences between the groups were statistically significant. Principal component analysis (PCA) was used to reveal the differences among sediments collected in different treatment microcosms. Redundancy analysis (RDA) and Pearson correlation analysis were used to assess the effects of environmental factors on the bacterial community and functional groups, including (1) the relationship between environmental variables and dominant bacteria at the genus and class level, (2) the relationship between functional groups and dominant bacteria, and (3) the relationship between functional groups and environmental variables (*n* = 6). For all tests, an *α* value of 0.05 was used to determine the significance of differences. To control for false positives, all *p* values for multiple comparisons were corrected using the false discovery rate (FDR < 0.05). All analyses were performed using R studio (v3.6.0) or STAMP software (v2.1.3) (Parks et al. 2014).

## Results

### Variation of physiochemical properties in sediment microenvironments

**Fig. 1:**
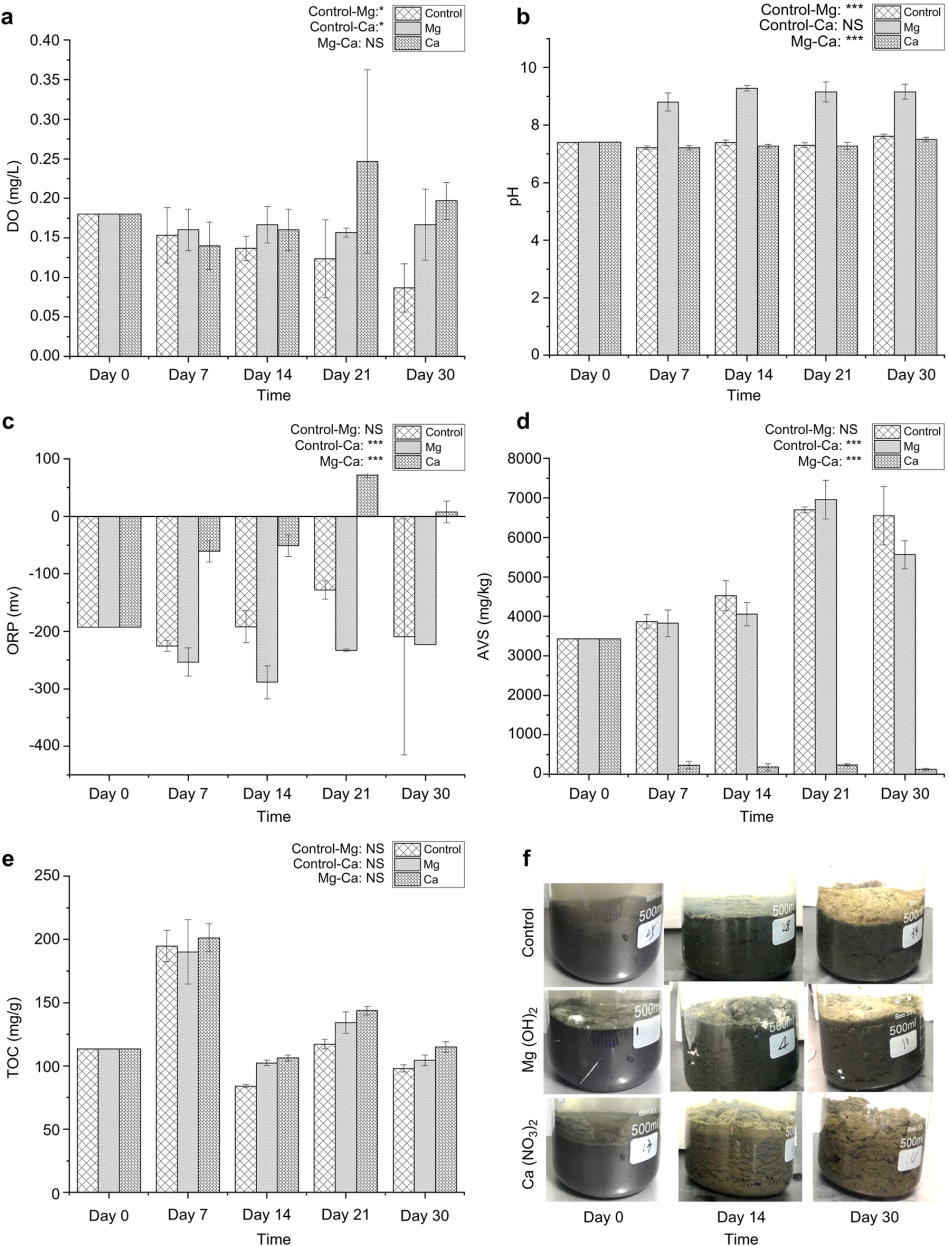
Changes in DO (**a**), pH (**b**), ORP (**c**), AVS (**d**), TOC (**e**), and appearance condition (**f**) in the control, Mg, and Ca microcosms during the experiment Error bars denote standard deviations (*n* = 9). Significant differences among the three microcosms were tested with Welch’s two-sample *t* test (*n* = 12, *α* = 0.05). Significance level: *p < 0.05, **p < 0.01, ***p < 0.001. *DO* dissolved oxygen, *ORP* oxidation/reduction potential, *AVS* acid volatile sulfide, *TOC* total organic carbon

Hereafter, Mg(OH)_2_ and Ca(NO_3_)_2_ treatments are referred to as Mg and Ca microcosms, respectively. The DO concentration in the Ca microcosms gradually increased, peaking on day 21, then decreased, whereas the DO concentration in the Mg microcosms remained stable at approximately 0.16 mg/L. The DO concentration in the control microcosms continuously decreased throughout the experimental period (Fig. 1a). The pH in the control and Ca microcosms remained neutral and exhibited no significant difference between them, whereas the pH in the Mg microcosms was significantly increased (*p* < 0.01), peaking at 9.28 on day 14, then remained alkaline (pH > 9) until day 30 (Fig. 1b). The ORP values in the control and Mg microcosms remained negative throughout the experimental period, whereas those in the Ca microcosms increased significantly (*p* < 0.01) from negative to positive and peaked at 72.1 mV on day 21 (Fig. 1c). The AVS concentration in the Ca microcosms was significantly decreased (*p* < 0.01) and remained low after day 7, whereas those in the control and Mg microcosms continuously increased and peaked on day 21; this was followed by a decrease in the Mg microcosms on day 30 (Fig. 1d). The TOC content in the control, Mg, and Ca microcosms showed similar variation, and was relatively higher in the Mg and Ca microcosms than in control microcosms (Fig. 1e). The sediment became fluffy and floated upward in the Ca microcosms, and the color changed from black to yellowish brown after the 30-day treatment. However, sediments in the Mg microcosms were much denser and showed no signs of floating upward, and only the surface sediment changed to yellowish brown (Fig. 1f).

### Sediment bacterial diversity and community composition

The sequencing results are presented in Table 1. The number of effective sequences ranged from 40,488 to 68,986, the number of OTUs with 97% similarity ranged from 1597 to 1825, and the coverage of all samples was above 0.970, indicating that the sequencing results accurately expressed the microorganism diversity. The rarefaction curves of all samples showed an upward trend, revealing a high OTU richness and abundance (Fig. S1). Although the highest Chao1 and ACE indices were obtained in the Ca microcosms on day 14, the overall Chao1 and ACE indices (including days 14 and 30) remained relatively higher in the Mg microcosms, indicating a higher richness of bacterial community after the 30-day treatment. The Shannon and Simpson indices of the Ca and Mg microcosms both decreased after the 30-day treatment, and exhibited no significant differences, which indicated that both Mg(OH)_2_ and Ca(NO_3_)_2_ addition treatments reduced the diversity of the sediment bacterial community and promoted the transformation of dominant bacteria. As shown in Fig. S2a, the results of the PCA at the genus level reveal significant differences in the microbial communities of the original sediments and control, Ca, and Mg microcosms (ADONIS, *R*^2^ = 0.114, *p* = 0.001). In particular, a clear separation between the community composition present in Ca microcosms and the other microcosms can be observed on the PC1 and PC2 axes (ADONIS, *R*^2^ = 0.083, *p* = 0.001). Furthermore, the composition of control, Ca, and Mg microcosms varied across treatment time (ADONIS, Origin vs Control30, *R*^2^ = 0.116, *p* = 0.001; Ca14 vs Ca30, *R*^2^ = 0.152, *p* = 0.001; Mg14 vs Mg30, *R*^2^ = 0.109, *p* = 0.001).

**Table 1:** Relative abundance and diversity data of the sediment bacterial community

Group	Sequence number	OTUs (97%)	Bacterial richness		Bacterial richness		Coverage
			Chao1	ACE	Shannon	Simpson	
Origin0	46,058 ± 58	1.696 ± 32***	2.082 ± 82	2.099 ± 70^**^	6.076 ± 0.064	0.006 ± 0.001	0.979 ± 0.002
Control30	26,214 ± 91***	1.604 ± 34	1.976 ± 96	2.010 ± 32	5.791 ± 0.032***	0.011 ± 0.002***	0.979 ± 0.003***
Mg14	49,731 ± 63*	1.739 ± 64	2041 ± 10	2.029 ± 56	6.204 ± 0.023***	0.006 ± 0.001**	0.982 ± 0.002***
Mg30	21,861 ± 94***	1.662 ± 24**	2.038 ± 108**	2.038 ± 12	6.013 ± 0.047	0.007 ± 0.000	0.980 ± 0.001
Ca14	51,458 ± 70**	1.825 ± 51***	2.162 ± 62***	2.160 ± 53***	6.239 ± 0.075***	0.005 ± 0.001***	0.980 ± 0.001
Ca30	60,391 ± 82***	1.597 ± 93***	1.843 ± 74***	1.870 ± 70***	6.002 ± 0.046	0.006 ± 0.002	0.983 ± 0.004

*OTUs* operational taxonomic units, *ACE* abundance-based coverage estimator

Significance levels: *p < 0.05, **p < 0.01, ***p < 0.001

**Fig. 2:**
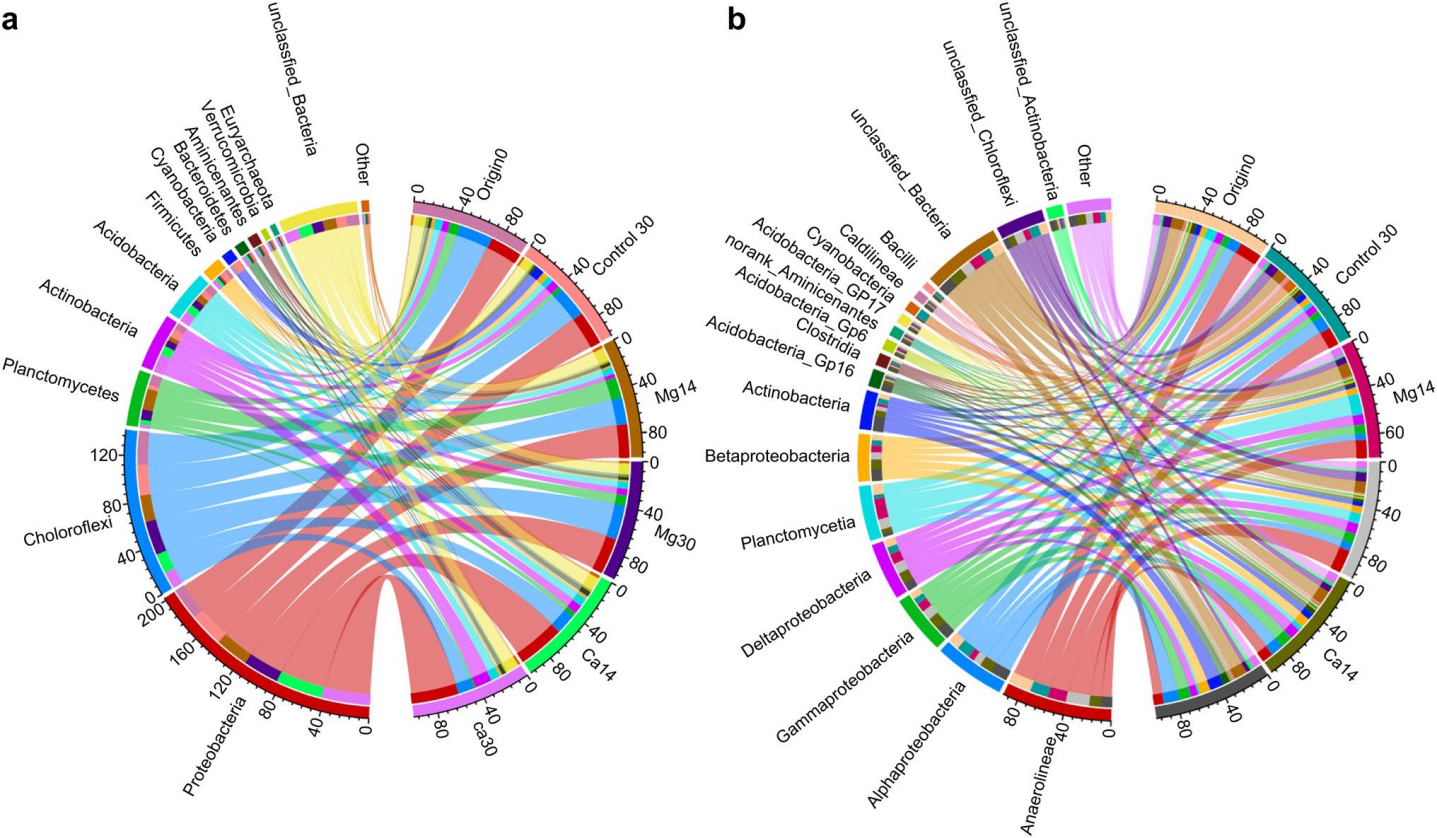
Community composition and abundance for different treatments at the phylum (**a**) and class (**b**) levels of Bacteria and Archaea Phyla and classes with average relative abundances less than 1.0% are grouped as Other

According to the OTU taxonomic analysis, 31 phyla within bacteria and archaea were identified, 11 of which had relative abundance greater than 1.0% in (Fig. 2a). In general, Proteobacteria was the most abundant phylum across all samples (average relative abundance of 33.68%), followed by Chloroflexi (23.45%), Planctomycetes (7.75%), and Actinobacteria (7.68%) (Fig. 2a; Table S1-1). Proteobacteria and Actinobacteria showed high relative abundance in the Ca microcosms (41.30 and 11.50%, respectively), whereas Chloroflexi and Planctomycetes were abundant in the Mg microcosms (26.57 and 13.73%, respectively) (Table S1-1). In addition, Cyanobacteria showed a high relative abundance in the control microcosms on day 30 (Fig. 2a; Table S1-1).

At the class level, a total of 79 classes were obtained across all samples, with 15 exhibiting a relative abundance of more than 1.0% (Fig. 2b). Anaerolineae was the most abundant class in all treatments, with an average relative abundance of 15.47%, followed by Alphaproteobacteria (9.97%), Gammaproteobacteria (8.28%), Deltaproteobacteria (8.03%), Planctomycetia (7.73%), Betaperoteobacteria (6.67%), and Actinobacteria (5.44%) (Fig. 2b; Table S1-2). The classes Anaerolineae and Planctomycetia were obviously more abundant in the Mg microcosms, whereas Alphaproteobacteria, Gammaproteobacteria, Betaproteobacteria, and Actinobacteria were more abundant in the Ca microcosms (Fig. 2a, b; Table S1-1 and S1-2).

**Fig. 3:**
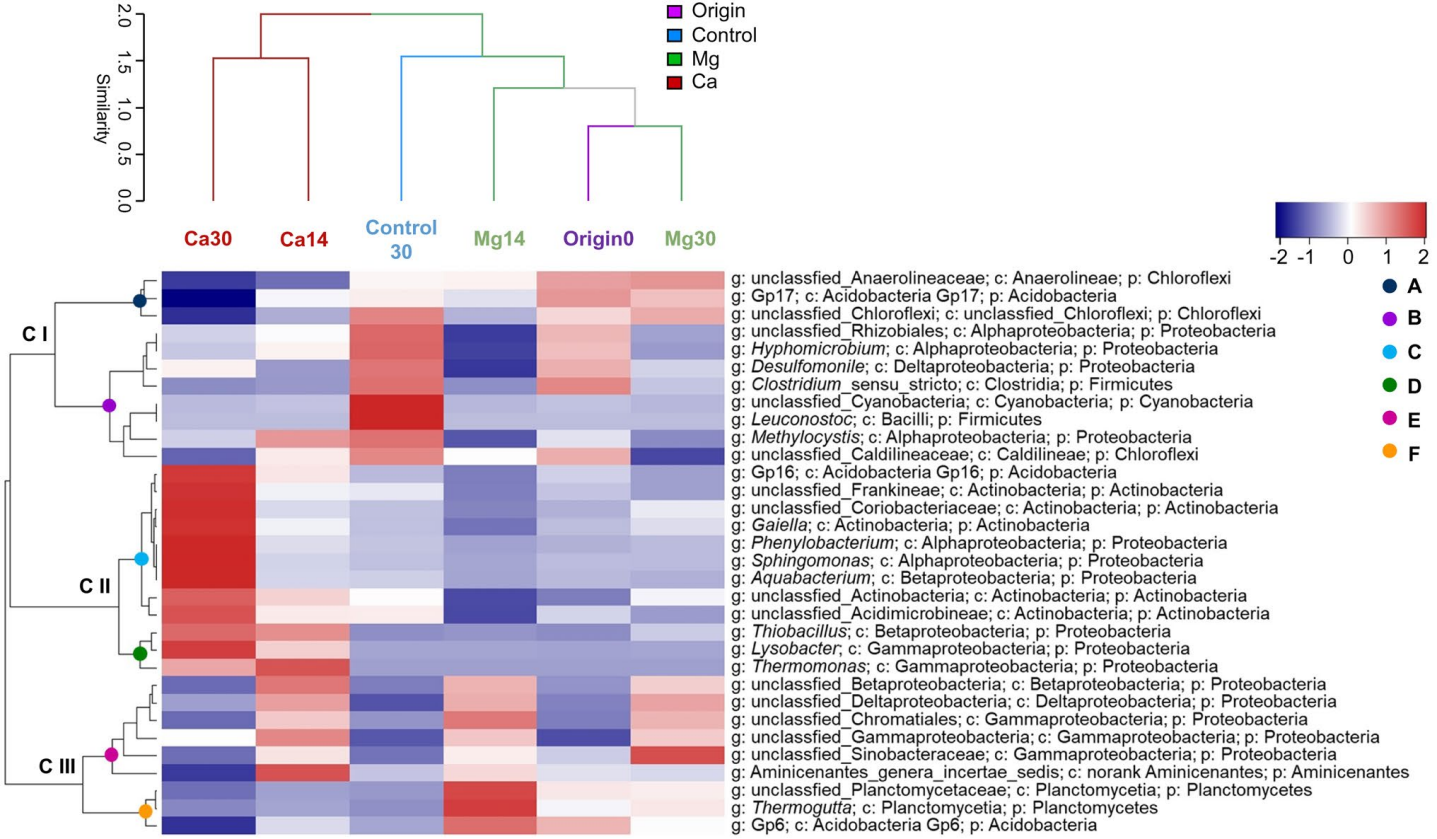
Bacterial community composition at the genus level in different samples shown by an abundance heatmap with clustering analysis Each row and column of the heatmap represents the genus (phylum/class/genus) and sample (treatment + day), respectively. Colors indicate the relative abundance from high (red) to low (blue). C I–III: cluster I–III; Letters A–F: subgroups

The 32 dominant genera, which included 15 unclassified genera, constituted more than 0.1% of the average relative abundance. An unclassified genus in the family Anaerolineaceae was dominant in all samples (average relative abundance of 14.43%), followed by unclassified genera in the family Planctomycetaceae (6.72%) and phylum Chloroflexi (6.68%) (Fig. 3; Table S1-3). Hierarchical clustering of samples at the genus level showed that, although the Mg microcosms on days 14 and 30 were clustered together with the original samples (day 0), the Ca microcosms on days 14 and 30 were clustered into an independent branch that was separate from the other microcosms (vertical dendrogram in Fig. 3).

The 32 dominant genera were divided into three clusters (horizontal dendrogram in Fig. 3). The genera in cluster I were dominant in the control microcosm on day 30, those in cluster II were dominant only in the Ca microcosms on day 30, and those in cluster III were dominant in the Mg microcosms on days 14 and 30 and in the Ca microcosms on day 14 (heatmap in Fig. 3). Clusters I, II, and III were further divided into subgroups A and B, subgroups C and D, and subgroups E and F, respectively. Subgroup A included genus Gp17 and two unclassified genera in phylum Chloroflexi, which were relatively abundant in the Mg microcosms on day 30. Subgroup B included unclassified genera in the classes Cyanobacteria, *Leuconostoc*, *Methylocystis*, and *Desulfomonile*, which exhibited relatively high abundance in the control microcosms (Control30) (Fig. 3, Table S1-3). Subgroup C genera that were abundant in the Ca microcosms on day 14 or 30 included the genera Gp16, *Gaiella*, and unclassified genera in the class Actinobacteria, whereas those in subgroup D included the genera *Thiobacillus*, *Lysobacter,* and *Thermomonas*. Subgroup E mainly consisted of *Aminicenantes* genera incertae sedis and other unclassified genera belonging to class Betaproteobacteria, Deltaproteobacteria, and Gammaproteobacteria, all of which were abundant in the Mg microcosms on days 14 and 30 and in the Ca microcosms on day 14. Subgroup F included the genera *Thermogutta*, Gp6, and unclassified genera belonging to the class Planctomycetia, which were mainly abundant in the Mg microcosms on day 14 (Fig. 3, Table S1-3).

### Variations in SRB and NRB composition

The composition of SRB and NRB detected by *dsrB* and *nirS* gene sequencing is shown in Fig. S3. The dominant species of SRB mainly belonged to the phyla Proteobacteria and Acidobacteria, the classes Acidobacteria and Deltaproteobacteria, and the genera *Desulfobacca*, *Desulfobulbus*, *Desulfomonile*, *Desulfosarcina*, and *Syntrophobacter* (Fig. S3a–c). Furthermore, the abundance of the genera *Desulfobacca*, *Desulfomonile*, and *Desulfosarcina* decreased in both Mg and Ca microcosms, whereas the genus *Syntrophobacter* decreased only in the Mg microcosms, and the genus *Desulfobacterium* decreased only in the Ca microcosms after the 30-day treatment. The dominant species of NRB mainly belonged to the phyla Proteobacteria and Planctomycetes, the classes Alphaproteobacteria, Betaproteobacteria, Gammaproteobacteria, and Planctomycetia, and the genera *Acidovorax*, *Dechlorononas*, *Dechlorospirillum*, *Pseudomonas*, *Rhodanobacter*, and *Rubrivivax* (Fig. S3d–f). Furthermore, the genera *Dechlorononas* and *Rhodanobacter* decreased in both Mg and Ca microcosms, the genera *Dechlorospirillum* and *Pseudomonas* decreased in the Mg microcosms and increased in the Ca microcosms, and the genus *Acidovorax* increased only in the Ca microcosms after the 30-day addition treatment.

### FAPROTAX and PICRUSt functional prediction of bacterial communities

Based on the FAPROTAX analysis, more than 90 functional groups were predicted to appear in all samples, and 23 functional groups with a relative abundance of greater than 0.1% were regarded as the dominant groups (Fig. S4a). Chemoheterotrophy and aerobic chemoheterotrophy (average relative abundance of 7.47% and 3.40%, respectively) were the most dominant functional groups in all treatments, followed by fermentation (2.19%), sulfate respiration (2.48%), and the respiration of sulfur compounds (2.49%) (Table S2). Fermentation and chloroplasts related to the carbon cycle were dominant in the control microcosms after the 30-day treatment (Fig. S4a). The abundance of functional groups related to sulfur and nitrogen cycles differed significantly between the control, Ca, and Mg microcosms. Sulfate respiration and respiration of sulfur compounds were decreased, whereas dark oxidation of sulfur compounds and dark sulfide oxidation were increased in the Ca microcosms after the 30-day treatment. Nitrogen fixation was obviously decreased in the Mg microcosms, and nitrate reduction was increased in the Ca microcosms (Fig. S4a). The differences in potential microbial ecological function, based on FAPROTAX prediction, among the samples from original sediment, control, Ca, and Mg microcosms are shown in a PCA plot (Fig. S2b). Samples of original sediment and control, Ca, and Mg microcosms are clearly distinct on both the PC1 and PC2 axes. The composition of potential microbial ecological function in the original sediment and the Mg microcosms vary from those of control and Ca microcosms (ADONIS, *R*^2^ = 0.129, *p* = 0.001), and the composition of potential microbial ecological function in the Ca microcosm also varied from those of original sediment, control, and Mg microcosms (ADONIS, *R*^2^ = 0.067, *p* = 0.002).

Based on the PICRUSt prediction, a total of six biological metabolic pathways (category level 1) were detected, including environmental information processing, metabolism, genetic information processing, cellular processes, organismal systems, and unclassified metabolic pathways, as well as 40 subfunctions (category level 2); the top 25 according to their relative abundance are shown in Fig. S4b. The relative abundance was highest for the genes potentially involved in the metabolism pathway in each treatment; the genes involved in the metabolism pathway subfunctions, including carbohydrate metabolism, amino acid metabolism, energy metabolism, metabolism of cofactors and vitamins, lipid metabolism, nucleotide metabolism, xenobiotics biodegradation, and metabolism, were also abundant (Fig. S4b). The genes involved in genetic information processing and environmental information processing pathways followed the metabolism pathway, and their subfunctions, including membrane transport, replication and repair, and translation, were also abundant in all treatments (Fig. S4b). The differences in potential microbial ecological function, based on PICRUSt prediction, among samples from original sediment and control, Ca, and Mg microcosms are shown in a PCA plot (Fig. S2c). Samples of the original sediment and Mg microcosm are distinct from the control and Ca microcosms on the PC1 axis, where the variation in the composition of potential microbial ecological function can be seen (ADONIS, *R*^2^ = 0.074, *p* = 0.001). Ca microcosm samples are clearly distinct from those of original sediment, control, and Mg microcosms on the PC2 axis. Furthermore, the composition of potential microbial ecological function in the Ca microcosm varied from the others (ADONIS, *R*^2^ = 0.078, *p* = 0.001).

Furthermore, PICRUSt predicted the occurrence of several genes potentially involved in nitrogen and sulfur metabolism subfunctions. The dissimilatory sulfate reduction (including genes encoding sulfate adenylyltransferase (*sat*), adenylylsulfate reductase subunit A/B (*aprAB*), and sulfate reductase dissimilatory-type alpha/beta subunit (*dsrAB*)) were obviously decreased in the Ca microcosms after the 30-day treatments, whereas the assimilatory sulfate reduction (including genes encoding sulfate adenylyltransferase subunit 2 (*cysD*), adenylylsulphate kinase (*cysC*), sulfite reductase (NADPH) flavoprotein alpha-component (*cysJ*), and sulfite reductase (NADPH) hemoprotein beta-component (*cysI*)) were obviously decreased in the Mg microcosms (Fig. S5a). Denitrification, including genes encoding nitrate reductase 1 alpha/beta subunit (*narG*/*narH*), nitrite reductase (NO-forming) (*nirK*, *nirS*), nitric oxide reductase cytochrome b-containing subunit II (*norB*), and nitrous-oxide reductase (*nosZ*)), were more abundant in the Ca microcosms than the Mg microcosms, whereas dissimilatory nitrate reduction to ammonium (including genes encoding periplasmic nitrate reductase NapA (*napA*), nitrite reductase (NAD(P)H) large/small subunit (*nirB/nirD*), and formate-dependent nitrite reductase periplasmic cytochrome c552 subunit (*nrfA*)) were relatively abundant in the Ca microcosms (Fig. S5b).

### Correlation between dominant bacterial genera and predicted metabolism functional groups

**Fig. 4:**
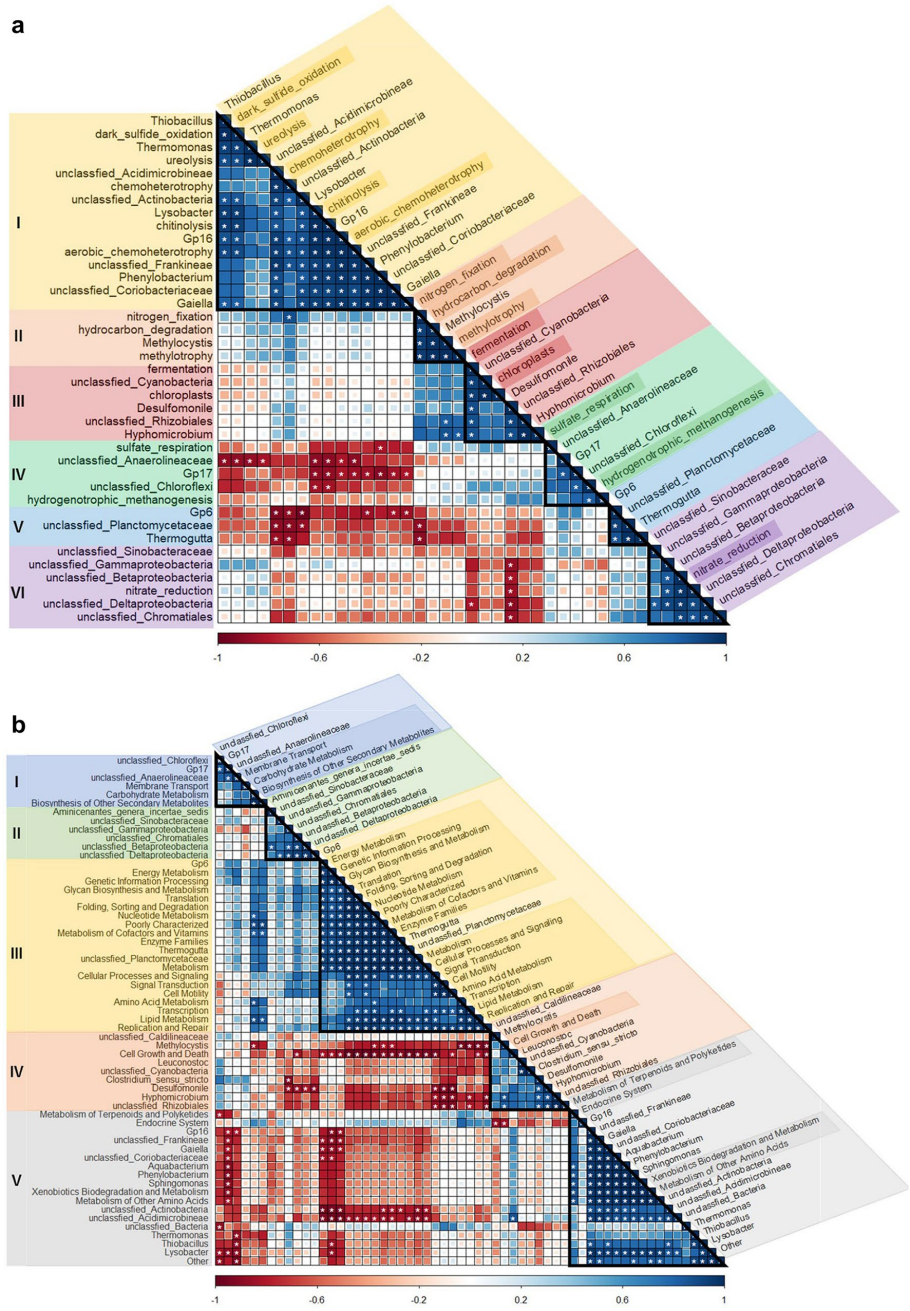
Heatmap of the Pearson correlation between dominant genera and functional groups based on FAPROTAX (**a**) and PICRUSt (**b**) predictions Size and darkness of color in each grid represent the level of the correlation coefficient. Blue and red colors represent positive and negative correlations, respectively. White asterisks indicate a coefficient and *p* value of ≥ 0.80 and < 0.05, respectively

Each bacterial functional group correlated well with certain dominant bacterial genera. The dominant genera and metabolic functional groups predicted by FAPROTAX were divided into six groups according to the Pearson correlation (Fig. 4a). Group I included the functional groups of chemoheterotrophy, aerobic chemoheterotrophy, chitinolysis, dark sulfide oxidation, and ureolysis, which exhibited a positive correlation with genera *Thiobacillus, Thermomonas*, *Lysobacter*, Gp16, *Phenylobacterium*, and *Gaiella*, as well as unclassified genera belonging to the phylum Actinobacteria. Group II included the functional groups of nitrogen fixation, hydrocarbon degradation, and methylotrophy, which were positively correlated with the genus *Methylocystis* (*p* < 0.05). Group III included the functional groups of fermentation and chloroplasts, which were positively correlated with the genera *Desulfomonile*, *Hyphomicrobium*, and unclassified genera belonging to the phyla Cyanobacteria and Proteobacteria. Group IV included the functional groups of sulfate respiration and hydrogenotrophic methanogenesis, which exhibited a positive correlation with Gp17 and unclassified genera in the phylum Chloroflexi. No functional groups were clustered into Group V, although the genera Gp6, *Thermogutta*, and unclassified genera belonging to phylum Planctomycetes were negatively correlated with the functional groups of chemoheterotrophy and nitrogen fixation (*p* < 0.05). Group VI included the functional groups of nitrate reduction, which were positively correlated with unclassified genera belonging to classes Gammaproteobacteria, Betaproteobacteria, and Deltaproteobacteria (*p* < 0.05) (Fig. 4a; Table S3-1 and S3-2).

The dominant genera and potential functions of sediment bacterial communities based on PICRUSt prediction were divided into five groups according to the Pearson correlation (Fig. 4b). Group I included the genes potentially involved in membrane transport, carbohydrate metabolism, and biosynthesis of other secondary metabolites, which showed a positive correlation with genus Gp17 and unclassified genera belonging to the phyla Chloroflexi and Anaerolineaceae. These genera were negatively correlated with the genes potentially involved in the metabolism of terpenoids and polyketides, the endocrine system, xenobiotic biodegradation, metabolism, and the metabolism of other amino acids (*p* < 0.05). No genes involved in potential functions were clustered into Group II, although the genus *Aminicenantes* genera incertae sedis, unclassified genera belonging to the family Sinobacteraceae, and classes Gammaproteobacteria, Betaproteobacteria, Deltaproteobacteria, and Chromatiales were negatively correlated with the genus *Desulfomonile* (*p* < 0.05) and the genes potentially involved in cell growth and death. Group III included genes involved in cellular processes, environmental information processing, genetic information processing, and metabolism, which were positively correlated with genera Gp6, *Thermogutta*, and unclassified genera belonging to the family Planctomycetaceae. These genera were significantly negatively correlated with the genes involved in cell growth and death (*p* < 0.05). Group IV included cell growth and death, and the genera *Methylocystis*, *Leuconostoc*, *Desulfomonile*, *Hyphomicrobium*, *Clostridium* sensu stricto, unclassified genera of family Caldilineaceae, phylum Cyanobacteria, and order Rhizobiales. Group V included genes potentially involved in the metabolism of terpenoids and polyketides, the endocrine system, xenobiotic biodegradation, metabolism, and the metabolism of other amino acids, which were positively correlated with the genus Gp16, *Gaiella*, *Aquabacterium*, *Phenylobacterium*, *Sphingomonas*, and unclassified genera belonging to the family Coriobacteriaceae, suborders Frankineae and Acidimicrobineae, and phylum Actinobacteria (*p* < 0.05) (Fig. 4b).

### Effects of sediment environmental factors on bacterial communities and metabolism functional groups

**Fig. 5:**
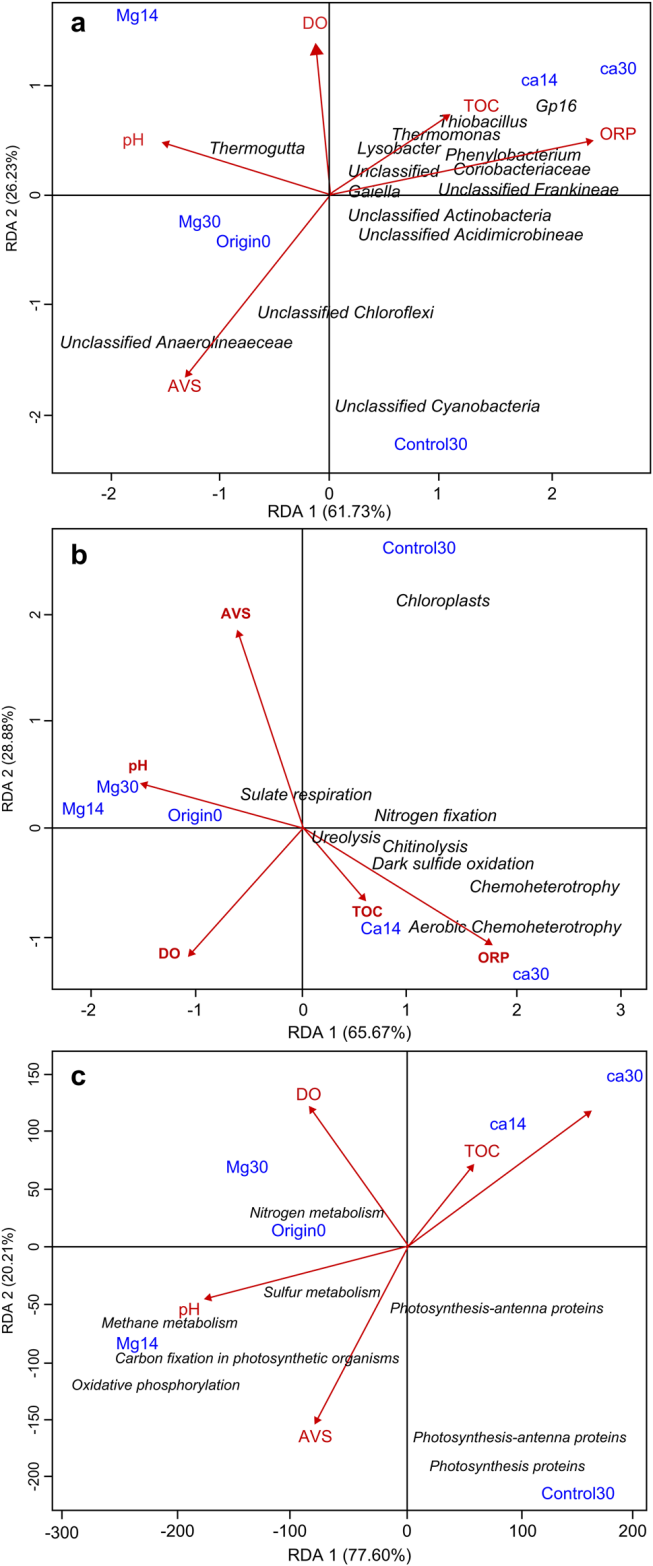
Redundancy analysis of the relationships between sediment environmental factors and **a** dominant genera, **b** metabolism functional groups based on FAPROTAX predictions, and **c** genes potentially involved in functional metabolisms based on PICRUSt predictions Only dominant genera, functional groups, and genes with *p* < 0.05 are shown. *RDA* redundancy analysis

According to the RDA, the overall variances of the bacterial community structures at the genus level were explained by 61.73% in RDA 1 and by 26.23% in RDA 2 (Fig. 5a), and the variances of the predicted metabolic functions were explained by 65.67 and 77.60% in RDA 1 and by 28.88 and 20.21% in RDA 2 (Fig. 5b, c). ORP was the principal environmental factor that explained the community variations in both diagrams (*p* < 0.05). Most of the dominant genera, including Gp16, *Gaiella*, *Thiobacillus*, *Thermomonas*, and *Lysobacter*, as well as the metabolism function groups, including chemoheterotrophy, aerobic chemoheterotrophy, dark sulfide oxidation, ureolysis, and chitinolysis, were positively correlated with ORP (*p* < 0.05), whereas unclassified genera in the family Anaerolineaceae, the functional groups of sulfate respiration, and the genes potentially involved in carbon fixation in photosynthetic organisms and oxidative phosphorylation were negatively correlated with ORP (*p* < 0.05) (Fig. 5a–c). Genes potentially involved in sulfur metabolism and methane metabolism were also negatively correlated with ORP (Fig. 5c). AVS was negatively correlated with genera *Thiobacillus*, *Thermomonas*, and *Lysobacter* and sulfide oxidation, ureolysis, and chitinolysis functional groups (*p* < 0.05), but positively correlated with unclassified genera in the phylum Chloroflexi and the sulfate respiration functional group (*p* < 0.05) (Fig. 5a, b). DO was negatively correlated with unclassified genera in the class Cyanobacteria and the chloroplast functional group, as well as genes potentially involved in photosynthesis-antenna proteins, photosynthesis, and photosynthesis protein functional groups (*p* < 0.05). pH was positively correlated with the genus *Thermogutta*, and potential genes involved in sulfur metabolism, methane metabolism, and oxidative phosphorylation, and negatively correlated with the nitrogen fixation functional group (*p* < 0.05) (Fig. 5a–c). TOC was positively correlated with the genus *Thermomonas* and the ureolysis functional group (*p* < 0.05) (Fig. 5a, b; Fig. S6 and S7).

## Discussion

### Effect of Mg(OH)_2_ and Ca(NO_3_)_2_ on the sediment redox state

The RDA results (Fig. 5) demonstrated that ORP and AVS were the two main environmental factors significantly affecting the majority of dominant bacteria and functional groups, as well as the Ca microcosm samples. The increased ORP and reduced AVS found in the Ca microcosms (Fig. 1) indicated that Ca(NO_3_)_2_ could effectively promote the redox state of sediment from reductive to oxidizing. Previous studies have reported that Ca(NO_3_)_2_, as a strong oxidant, can effectively improve the reduction state and inhibit the release of H_2_S in contaminated sediments through a series of biochemical reactions, including the decomposition and transformation of organic matter or reductive substances, as well as denitrification and sulfur-dependent autotrophic denitrification (Lau et al. 2017; Li et al. 2017; Pan et al. 2018; Boyd 2020). The genus *Thermomonas*, belonging to class Gammaproteobacteria, and genus *Thiobacillus,* belonging to class Betaproteobacteria, are well-known heterotrophic and sulfur-dependent autotrophic denitrification bacteria in freshwater sediments (Shao et al. 2010; Li et al. 2017; Han et al. 2019; Hao et al. 2020). The relative abundance of these two genera in the Ca microcosms was significantly increased, confirming that Ca(NO_3_)_2_ addition promoted the occurrence of denitrification and the reduction of black-odorous substances in sediments. Conversely, increased ORP and reduced AVS were only observed on day 30 in the Mg microcosms, indicating that the addition of Mg(OH)_2_ as a treatment method might require a more extensive treatment period before improving contaminated sediments. Although pH is a key factor affecting the activity of bacterial metabolism (Pepino Minetti et al. 2017; Yun et al. 2017; Liu et al. 2019; Chen et al. 2021), the RDA and Pearson correlation analysis in the present study showed a less significant correlation between pH and the dominant bacteria and functional groups, suggesting that the weak alkalinity induced by Mg(OH)_2_ was not sufficient to affect the activity of the bacterial community and their metabolic functions in contaminated sediments. Thus, combining Mg(OH)_2_ with other biochemical reagents might enhance the improvement effect of Mg(OH)_2_.

### Effects of Mg(OH)_2_ and Ca(NO_3_)_2_ addition on predicted bacterial metabolism functions

The composition and ecological functions of microbial communities in sediments are complex, and they vary in different environments (Tang et al. 2018; Liu et al. 2019; Chen et al. 2022; Yun et al. 2022). Sediment samples of the Mg microcosm collected on day 30 (Mg30) and original sediment samples (Origin) were found to have a close distribution in the PCA plots (Fig. S2), suggesting a similarity in microbial community composition and potential microbial ecological function between the original sediment and the Mg microcosm after 30 days of Mg(OH)_2_ addition treatment. Furthermore, samples of the Ca microcosm (Ca14 and Ca30) were distinct from those of the Mg microcosm on both the PC1 and PC2 axes (Fig. S2). This suggests a variation in microbial community and their potential functions as a result of the different treatments. Previous studies have reported that bacterial aerobic metabolism is stronger than anaerobic metabolism (Louca and Doebeli 2017), and nitrogen metabolism is more active than sulfur metabolism in the sediments of urban rivers and lakes (Li et al. 2019; Fu et al. 2020). PICRUSt functional prediction analysis of the present study also showed that the genes involved in nitrogen metabolism were more abundant than those involved in sulfur metabolism, and the genes involved in sulfur metabolism were relatively more abundant in Mg microcosms, indicating much more active sulfur metabolism under the Mg(OH)_2_ addition treatment.

Functional bacteria related to aerobic chemoheterotrophy, nitrogen fixation, and sulfide oxidation processes were abundant in the Ca microcosms (Figs. 3, 4a), indicating an obvious promotion effect on bacterial oxidation metabolism under the Ca(NO_3_)_2_ addition treatment. Indeed, (He et al. 2017, 2018 and Li et al. 2019 reported that the addition of Ca(NO_3_)_2_ could enhance the denitrification effect and the persistence of microbial autotrophic denitrification in sediments. In the present study, the genera *Thermomonas* and *Thiobacillus*, which belong to heterotrophic and sulfur-dependent autotrophic denitrification bacteria, were obviously increased under Ca(NO_3_)_2_ addition treatment, although their relative abundance only accounted for a small proportion of the total bacteria.

### Relationships between bacterial taxonomic groups and predicted metabolism functions

Among the 211 genera detected in this study, 36 genera were abundant in the Ca microcosms (Fig. S8). Bacteria that increased under Ca(NO_3_)_2_ treatment mainly belonged to the classes Alphaproteobacteria, Betaproteobacteria, Gammaproteobacteria, and Actinobacteria (Table S1-1), including the aerobic and chemotrophic genera *Sphingomonas* (class Alphaproteobacteria), *Thiobacillus* (class Betaproteobacteria), *Lysobacter* (class Gammaproteobacteria), and *Gaiella* (class Actinobacteria) (Table S1-2 and S1-3).

Most members of the class Alphaproteobacteria are chemoheterotrophic or chemoautotrophic bacteria and play significant roles in nitrogen fixation and the decomposition and assimilation of organic matter in organically enriched sediment (Chaudhary and Kim 2016; Ghosal et al. 2016; Fu et al. 2020; Sun et al. 2020; Zhang et al. 2021a, b). Similar results were also observed in the present study, whereby the dominant genera of class Alphaproteobacteria were abundant in the Ca microcosms and closely correlated with aerobic metabolism, which confirmed the contribution of Ca(NO_3_)_2_ to promoting the activity of aerobic and chemoheterotrophic metabolisms in the sediments.

The genus *Thiobacillus*, which belongs to class Betaproteobacteria, is a well-known sulfur-dependent autotrophic denitrification bacterium that can use nitrate as an electron acceptor and sulfide as an electron donor to achieve the goal of simultaneous desulfurization and denitrification (Di Capua et al. 2016; He et al. 2017; Chen et al. 2019). (Wang et al. 2016a, b; Hao et al. 2020; and Zhang et al. 2022) also reported that *Thiobacillus denitrificans* could be effectively used for desulfurization and denitrification in black-odorous sediments under Ca(NO_3_)_2_ treatment. Similarly, the results of the present study also showed that the genus *Thiobacillus* was abundant in the Ca microcosms and significantly positively correlated with the functional bacteria of dark sulfide oxidation, as well as the genes involved in denitrification and dissimilatory nitrate reduction to ammonium metabolism (these genes including *narG*, *nirK*, *nirS*, *norC*, *norB*, *nasB*, and *narL*) (*p* < 0.05) (Fig. S5, Fig. 4). Therefore, these results confirmed the improvement effect of Ca(NO_3_)_2_ treatment on the redox state of sediments and the desulfurization ability of genus *Thiobacillus* (Li et al. 2019; Huang et al. 2020).

Conversely, the bacteria that increased under Mg(OH)_2_ treatment mainly belonged to the classes Anaerolineae and Planctomycetia (Fig. 2b, Table S1-1), including the anaerobic unclassified genera in class Anaerolineae and genera *Thermogutta* in class Planctomycetia (Fig. 3; Table S1-2 and S1-3). Previous research has demonstrated that most known species in the class Anaerolineae are fermentative, syntrophic, and strictly anaerobic organisms (Yamada et al. 2006; Wang et al. 2016b; Cai et al. 2021; Ni et al. 2022) and have the capability to cooperatively degrade carbohydrates and alkanes with hydrogenotrophic methanogens (Friedl et al. 2018; Rahman et al. 2019; Li et al. 2020a, b). Pearson correlation analysis of bacterial taxonomic groups and their predicted metabolic functions also showed a significant positive correlation between unclassified genera of the family Anaerolineaceae and the hydrogenotrophic methanogenesis functional group (Fig. 4a), as well as the genes involved in energy metabolism (including methane metabolism and carbon fixation in photosynthetic organisms) (*p* < 0.05) (Fig. 4b). Furthermore, the significant negative correlation with aerobic chemoheterotrophy and dark sulfide oxidation (*p* < 0.05) (Fig. 4a) indicated a relatively stronger anaerobic state in the sediments of the Mg microcosms.

The phylum Cyanobacteria is the dominant nanophytoplankton community in the surface water of most eutrophic water areas and could also be the main species in sediments formed by vertical sinking (Fu et al. 2020). In the present study, the phylum Cyanobacteria and its positively correlated functional groups chloroplasts and fermentation, as well as the genes involved in photosynthesis proteins (*p* < 0.01), only increased in the control microcosms (Control30) after the 30-day treatment (Fig. 3; Fig. S4), whereas those in the Mg and Ca microcosms decreased significantly (*p* < 0.05). These results indicate that both Mg(OH)_2_ and Ca(NO_3_)_2_ could effectively inhibit the growth of several genera belonging to the phylum Cyanobacteria; therefore, they may be able to control and even inhibit the development of Cyanobacterial blooms and eutrophication in water ecosystems.

In conclusion, this study revealed the differences in sedimental properties and the richness, composition, and predicted potential functions of sediment bacterial communities before and after remediation. The addition of Ca(NO_3_)_2_ significantly enhanced the oxidation state in sediments and effectively promoted the growth and activity of functional bacteria related to aerobic metabolism, indicating that Ca(NO_3_)_2_ more rapidly improves the hypoxia-related reducing state in sediments than Mg(OH)_2_. Additionally, the addition of Mg(OH)_2_ significantly promoted the abundance and activity of correlated genes and bacteria involved in sulfur metabolism, carbon fixation metabolism, and methane metabolism, indicating that Mg(OH)_2_ accelerates carbon and sulfur metabolism in sediments. This study deepens the understanding of the remediation effect of these remediation reagents on microbial community composition and ecological function in sediment. However, the remediation effect on different types of polluted water bodies, effects of a concentration series of remediation reagents, and remediation mechanisms need to be further studied.

## Supplementary Information

Below is the link to the electronic supplementary material.

Supplementary file1 (DOCX 6280 KB)

## Data Availability

All data generated or analyzed during this study are included in this published article and its supplementary information files.
