# Supplementary material for: Effect of remediation reagents on bacterial composition and ecological function in black-odorous water sediments
Source: Arch Microbiol. 2022 Apr 24;204(5):280. doi: 10.1007/s00203-022-02871-4 (PMC9035426; doi:10.1007/s00203-022-02871-4)
Supplement: Supplementary file 1 — Supplementary file1 (DOCX 6280 KB) [file 203_2022_2871_MOESM1_ESM.docx]

**Supplementary Information**

**Effect of remediation reagents on bacterial composition and ecological function in black-odorous water sediments**

Journal name: *Archives of Microbiology*

Dong Xia^1,2^, Hanbin Zhao^1^, Sohei Kobayashi^1,2^, Qi Mi^3^, Aimin Hao^1,2^*, Yasushi Iseri^1,2^

^1^College of Life and Environmental Science, Wenzhou University, Wenzhou, Zhejiang 325035, China

^2^National and Local Joint Engineering Research Center of Ecological Treatment Technology for Urban Water Pollution, Wenzhou University, Wenzhou 325035, China

^3^Nanjing Guoxing Environmental Protection Industry Research Institute Co. LTD, Nanjing 211899, China

*Corresponding author: Aimin Hao

College of Life and Environmental Science, Wenzhou University

Wenzhou, Zhejiang 325035, China

Phone: (+86) 17357758606

Fax: (+86) 0577-86689079

Email: hao_aimin@wzu.edu.cn

**Supplementary Tables**

**Table S1-1** Relative abundance (%) of the dominant phyla detected in this study

| **Phylum** | **Origin0** | **Control30** | **Mg14** | **Mg30** | **Ca14** | **Ca30** | **Total-aver.** | **Mg-aver.** | **Ca-aver.** |  |
| --- | --- | --- | --- | --- | --- | --- | --- | --- | --- | --- |
| Proteobacteria | 28.37 | 29.93 | 29.29 | 31.88 | 41.00 | 41.61 | 33.68 | 30.58 | 41.30 | |
| Chloroflexi | 29.43 | 26.90 | 23.27 | 29.88 | 17.34 | 13.87 | 23.45 | 26.57 | 15.61 | |
| Planctomycetes | 9.48 | 3.68 | 18.33 | 9.13 | 4.10 | 1.80 | 7.75 | 13.73 | 2.95 | |
| Actinobacteria | 6.18 | 7.47 | 3.55 | 5.91 | 8.00 | 14.99 | 7.68 | 4.73 | 11.50 | |
| Acidobacteria | 6.62 | 5.30 | 6.07 | 6.01 | 7.27 | 8.18 | 6.57 | 6.04 | 7.73 | |
| Firmicutes | 2.93 | 5.22 | 1.57 | 1.48 | 2.66 | 2.36 | 2.70 | 1.53 | 2.51 | |
| Cyanobacteria | 0.47 | 7.75 | 0.16 | 0.14 | 0.54 | 0.39 | 1.58 | 0.15 | 0.46 | |
| Bacteroidetes | 1.08 | 0.87 | 1.42 | 1.48 | 2.03 | 1.99 | 1.48 | 1.45 | 2.01 | |
| Aminicenantes | 1.41 | 1.36 | 1.56 | 1.40 | 1.84 | 1.11 | 1.45 | 1.48 | 1.48 | |
| Verrucomicrobia | 1.02 | 0.58 | 1.31 | 0.63 | 0.69 | 0.58 | 0.80 | 0.97 | 0.64 | |
| Euryarchaeota | 1.07 | 0.94 | 0.29 | 1.21 | 0.80 | 0.42 | 0.79 | 0.75 | 0.61 | |
| Unclassified Bacteria | 11.07 | 9.24 | 11.77 | 9.86 | 12.31 | 11.89 | 11.02 | 10.81 | 12.10 | |
| Other | 0.87 | 0.77 | 1.42 | 0.99 | 1.42 | 0.80 | 1.04 | 1.21 | 1.11 | |

**Total-aver.:** average value of six sediment microcosms; **Mg-aver.:** average value of Mg(OH)_2_ treatment microcosms; **Ca-aver.:** average value of Ca(NO_3_)_2_ treatment microcosms.

**Table S1-2** Relative abundance (%) of the dominant classes detected in this study

| **Class** | **Origin0** | **Control30** | **Mg14** | **Mg30** | **Ca14** | **Ca30** | **Total-aver.** | **Mg-**  **aver.** | **Ca-**  **aver.** |
| --- | --- | --- | --- | --- | --- | --- | --- | --- | --- |
| Anaerolineae | 20.42 | 16.33 | 16.27 | 20.65 | 10.22 | 8.90 | 15.47 | 18.46 | 9.56 |
| Alphaproteobacteria | 10.13 | 12.20 | 5.37 | 7.01 | 11.06 | 14.03 | 9.97 | 6.19 | 12.54 |
| Gammaproteobacteria | 5.82 | 5.58 | 8.00 | 8.73 | 10.90 | 10.64 | 8.28 | 8.36 | 10.77 |
| Deltaproteobacteria | 6.93 | 6.51 | 9.22 | 9.28 | 9.65 | 6.56 | 8.03 | 9.25 | 8.11 |
| Planctomycetia | 9.46 | 3.65 | 18.30 | 9.11 | 4.07 | 1.80 | 7.73 | 13.70 | 2.93 |
| Betaproteobacteria | 4.86 | 4.98 | 5.96 | 6.23 | 8.49 | 9.46 | 6.67 | 6.10 | 8.98 |
| Actinobacteria | 4.39 | 5.16 | 1.97 | 3.66 | 5.49 | 11.96 | 5.44 | 2.81 | 8.73 |
| Acidobacteria Gp16 | 2.08 | 1.83 | 1.10 | 1.51 | 2.97 | 5.33 | 2.47 | 1.31 | 4.15 |
| Clostridia | 2.37 | 2.52 | 1.25 | 1.24 | 1.50 | 1.55 | 1.74 | 1.25 | 1.52 |
| Acidobacteria Gp6 | 1.89 | 1.29 | 2.17 | 1.58 | 1.46 | 0.89 | 1.55 | 1.88 | 1.18 |
| Norank Aminicenantes | 1.41 | 1.36 | 1.56 | 1.40 | 1.84 | 1.11 | 1.45 | 1.48 | 1.48 |
| Acidobacteria Gp17 | 1.80 | 1.51 | 1.36 | 1.66 | 1.43 | 0.73 | 1.41 | 1.51 | 1.08 |
| Cyanobacteria | 0.31 | 7.59 | 0.06 | 0.07 | 0.32 | 0.12 | 1.41 | 0.06 | 0.22 |
| Caldilineae | 1.43 | 1.60 | 1.20 | 0.91 | 1.36 | 1.09 | 1.26 | 1.05 | 1.23 |
| Bacilli | 0.52 | 2.69 | 0.28 | 0.21 | 1.14 | 0.73 | 0.93 | 0.25 | 0.94 |
| Unclassified Chloroflexi | 7.55 | 8.96 | 5.76 | 8.30 | 5.68 | 3.83 | 6.68 | 7.03 | 4.76 |
| Unclassified Actinobacteria | 1.79 | 2.31 | 1.59 | 2.25 | 2.50 | 3.03 | 2.24 | 1.92 | 2.77 |
| Unclassified Bacteria | 11.07 | 9.24 | 11.77 | 9.86 | 12.31 | 11.89 | 11.02 | 10.81 | 12.10 |
| Other | 5.78 | 4.70 | 6.81 | 6.32 | 7.60 | 6.34 | 6.26 | 6.56 | 6.97 |

**Total-aver.:** average value of six sediment microcosms; **Mg-aver.:** average value of Mg(OH)_2_ treatment microcosms; **Ca-aver.:** average value of Ca(NO_3_)_2_ treatment microcosms.

**Table S1-3** Relative abundance (%) of the dominant genera detected in this study

| **Genus** | **Origin0** | **Control**  **30** | **Mg14** | **Mg30** | **Ca14** | **Ca30** | **Total-aver.** | **Mg-aver.** | **Ca-**  **aver.** |
| --- | --- | --- | --- | --- | --- | --- | --- | --- | --- |
| Unclassified Anaerolineaceae | 19.02 | 15.32 | 15.44 | 19.47 | 9.62 | 7.74 | 14.43 | 17.46 | 8.68 |
| Unclassified Planctomycetaceae | 8.35 | 3.18 | 15.69 | 8.04 | 3.52 | 1.55 | 6.72 | 11.87 | 2.53 |
| Unclassified Chloroflexi | 7.55 | 8.96 | 5.76 | 8.30 | 5.68 | 3.83 | 6.68 | 7.03 | 4.76 |
| Unclassified Rhizobiales | 3.86 | 4.45 | 2.12 | 2.75 | 3.32 | 3.05 | 3.26 | 2.44 | 3.18 |
| Unclassified Betaproteobacteria | 3.81 | 3.74 | 4.46 | 4.35 | 4.69 | 3.68 | 4.12 | 4.41 | 4.19 |
| Unclassified Sinobacteraceae | 3.43 | 2.84 | 3.90 | 5.13 | 3.98 | 2.80 | 3.68 | 4.51 | 3.39 |
| *Desulfomonile* | 2.19 | 2.44 | 1.13 | 1.69 | 1.49 | 1.91 | 1.81 | 1.41 | 1.70 |
| Gp16 | 2.08 | 1.83 | 1.10 | 1.51 | 2.97 | 5.33 | 2.47 | 1.31 | 4.15 |
| Unclassified Deltaproteobacteria | 1.92 | 1.51 | 3.79 | 3.89 | 3.92 | 2.16 | 2.87 | 3.84 | 3.04 |
| Gp6 | 1.89 | 1.29 | 2.17 | 1.58 | 1.46 | 0.89 | 1.55 | 1.88 | 1.18 |
| Gp17 | 1.80 | 1.51 | 1.36 | 1.66 | 1.43 | 0.73 | 1.41 | 1.51 | 1.08 |
| Unclassified Actinobacteria | 1.79 | 2.31 | 1.59 | 2.25 | 2.50 | 3.03 | 2.24 | 1.92 | 2.77 |
| Aminicenantes genera incertae sedis | 1.41 | 1.36 | 1.56 | 1.40 | 1.84 | 1.11 | 1.45 | 1.48 | 1.48 |
| *Methylocystis* | 1.31 | 2.48 | 0.47 | 0.78 | 2.22 | 1.20 | 1.41 | 0.62 | 1.71 |
| *Clostridium* sensu stricto | 1.13 | 1.22 | 0.27 | 0.46 | 0.30 | 0.27 | 0.61 | 0.36 | 0.28 |
| Unclassified Gammaproteobacteria | 1.13 | 1.15 | 1.72 | 1.71 | 1.91 | 1.56 | 1.53 | 1.72 | 1.74 |
| Unclassified Acidimicrobineae | 1.06 | 1.41 | 0.32 | 0.75 | 1.42 | 2.39 | 1.23 | 0.54 | 1.91 |
| Unclassified Caldilineaceae | 1.06 | 1.13 | 0.91 | 0.63 | 0.95 | 0.67 | 0.89 | 0.77 | 0.81 |
| *Hyphomicrobium* | 0.92 | 1.07 | 0.52 | 0.65 | 0.83 | 0.72 | 0.79 | 0.59 | 0.78 |
| Unclassified Frankineae | 0.81 | 1.02 | 0.38 | 0.59 | 1.07 | 2.61 | 1.08 | 0.48 | 1.84 |
| Unclassified Coriobacteriaceae | 0.64 | 0.71 | 0.44 | 0.89 | 0.82 | 2.06 | 0.93 | 0.67 | 1.44 |
| *Thermogutta* | 0.64 | 0.20 | 1.67 | 0.80 | 0.29 | 0.16 | 0.63 | 1.24 | 0.22 |
| Unclassified Chromatiales | 0.60 | 0.66 | 1.39 | 1.19 | 1.13 | 0.54 | 0.92 | 1.29 | 0.83 |
| *Gaiella* | 0.50 | 0.52 | 0.21 | 0.63 | 0.71 | 1.74 | 0.72 | 0.42 | 1.23 |
| Unclassified Cyanobacteria | 0.28 | 7.57 | 0.06 | 0.06 | 0.32 | 0.12 | 1.40 | 0.06 | 0.22 |
| *Phenylobacterium* | 0.19 | 0.33 | 0.07 | 0.27 | 0.52 | 2.73 | 0.69 | 0.17 | 1.62 |
| *Sphingomonas* | 0.12 | 0.13 | 0.05 | 0.11 | 0.19 | 1.13 | 0.29 | 0.08 | 0.66 |
| *Aquabacterium* | 0.10 | 0.18 | 0.02 | 0.06 | 0.21 | 1.35 | 0.32 | 0.04 | 0.78 |
| *Thiobacillus* | 0.06 | 0.07 | 0.11 | 0.44 | 1.57 | 1.84 | 0.68 | 0.28 | 1.71 |
| *Thermomonas* | 0.00 | 0.00 | 0.00 | 0.00 | 1.73 | 1.17 | 0.48 | 0.00 | 1.45 |
| *Lysobacter* | 0.00 | 0.01 | 0.01 | 0.01 | 0.82 | 1.78 | 0.44 | 0.01 | 1.30 |
| *Leuconostoc* | 0.00 | 1.72 | 0.00 | 0.00 | 0.00 | 0.00 | 0.29 | 0.00 | 0.00 |
| **unclassfied_Bacteria** | 11.07 | 9.24 | 11.77 | 9.86 | 12.31 | 11.89 | 11.02 | 10.81 | 12.10 |
| **Other** | 19.28 | 18.47 | 19.53 | 18.06 | 24.25 | 26.27 | 20.98 | 18.80 | 25.26 |

**Total-aver.:** average value of six sediment microcosms; **Mg-aver.:** average value of Mg(OH)_2_ treatment microcosms; **Ca-aver.:** average value of Ca(NO_3_)_2_ treatment microcosms.

**Table S2** Abundance of the dominant functional groups of bacterial communities determined through FAPROTAX function prediction analysis

| **Functional Group** | | **Origin** | **Control30** | **Mg14** | **Mg30** | **Ca14** | **Ca30** |
| --- | --- | --- | --- | --- | --- | --- | --- |
| Chemoheterotrophy | | 1,844 | 2,685 | 1,182 | 1,331 | 2,416 | 3,146 |
| Aerobic chemoheterotrophy | | 749 | 838 | 598 | 590 | 1,186 | 1,776 |
| Sulfur  cycle | Respiration of sulfur compounds | 672 | 733 | 696 | 711 | 706 | 614 |
|  | Sulfate respiration | 672 | 733 | 688 | 704 | 705 | 613 |
|  | Dark oxidation of sulfur compounds | 14 | 24 | 25 | 101 | 371 | 439 |
|  | Dark sulfide oxidation | 13 | 16 | 24 | 97 | 344 | 403 |
| Carbon cycle | Photoheterotrophy | 32 | 25 | 36 | 24 | 44 | 37 |
|  | Phototrophy | 32 | 25 | 36 | 24 | 44 | 37 |
|  | Chloroplasts | 103 | 1,694 | 35 | 31 | 117 | 86 |
|  | Methylotrophy | 422 | 631 | 256 | 282 | 629 | 405 |
|  | Methanotrophy | 340 | 603 | 245 | 257 | 602 | 363 |
|  | Fermentation | 680 | 1,214 | 328 | 459 | 422 | 581 |
|  | Hydrocarbon degradation | 340 | 603 | 245 | 257 | 602 | 363 |
|  | Aromatic compound degradation | 10 | 86 | 4 | 9 | 21 | 161 |
|  | Methanogenesis | 234 | 201 | 64 | 264 | 171 | 92 |
|  | Methanogenesis by CO_2_ reduction | 181 | 143 | 36 | 147 | 88 | 47 |
|  | Hydrogenotrophic methanogenesis | 197 | 161 | 40 | 169 | 106 | 53 |
|  | Ureolysis | 108 | 51 | 51 | 27 | 405 | 271 |
|  | Chitinolysis | 0 | 2 | 2 | 3 | 180 | 389 |
| Nitrogen cycle | Nitrogen fixation | 325 | 594 | 113 | 197 | 533 | 455 |
|  | Nitrate reduction | 146 | 148 | 185 | 168 | 197 | 150 |
|  | Nitrate respiration | 146 | 146 | 185 | 166 | 194 | 139 |
|  | Nitrogen respiration | 146 | 146 | 185 | 166 | 194 | 139 |
| Other | | 19,784 | 17,477 | 20,343 | 20,105 | 18,700 | 18,459 |
| **Total** | | 27,190 | 28,979 | 25,602 | 26,289 | 28,977 | 29,218 |

Functional groups with an average abundance (sequence number) of less than 30 were grouped as “Other.”

**Table S3-1** Pearson correlation between dominant genera and functional groups

| **Functional group** | **Gp16** | **Gp6** | **Gp17** | **Desu** | **Methy** | **Hypho** | **Pheny** | **Sphi** | **Thio** | **Aqua** | **monas** | **Lyso** |
| --- | --- | --- | --- | --- | --- | --- | --- | --- | --- | --- | --- | --- |
| Chemoheterotrophy | 0.818* | -0.883* | -0.621 | 0.548 | 0.646 | 0.556 | 0.740 | 0.722 | 0.627 | 0.760 | 0.575 | 0.737 |
| Aerobic chemoheterotrophy | 0.988*** | -0.789 | -0.863* | 0.100 | 0.214 | 0.042 | 0.941** | 0.929** | 0.894* | 0.938** | 0.766 | 0.985*** |
| Respiration of sulfur compounds | -0.968** | 0.776 | 0.726 | -0.347 | -0.299 | -0.259 | -0.895* | -0.892* | -0.759 | -0.905* | -0.643 | -0.895* |
| Sulfate respiration | -0.977*** | 0.773 | 0.751 | -0.314 | -0.261 | -0.214 | -0.912** | -0.911** | -0.773 | -0.922** | -0.646 | -0.911* |
| Thiosulfate respiration | -0.520 | 0.560 | 0.100 | -0.743 | -0.782 | -0.849* | -0.333 | -0.326 | -0.300 | -0.364 | -0.390 | -0.362 |
| Sulfur respiration | -0.520 | 0.560 | 0.100 | -0.743 | -0.782 | -0.849* | -0.333 | -0.326 | -0.300 | -0.364 | -0.390 | -0.362 |
| Sulfite respiration | 0.630 | -0.228 | -0.807 | -0.184 | -0.480 | -0.447 | 0.755 | 0.778 | 0.401 | 0.768 | 0.178 | 0.653 |
| Dark oxidation of sulfur compounds | 0.869* | -0.695 | -0.739 | -0.189 | 0.182 | -0.139 | 0.781 | 0.756 | 1.000*** | 0.750 | 0.926** | 0.935** |
| Dark sulfide oxidation | 0.862* | -0.684 | -0.732 | -0.203 | 0.169 | -0.153 | 0.774 | 0.749 | 1.000*** | 0.742 | 0.926** | 0.931** |
| Chloroplasts | -0.181 | -0.298 | 0.124 | 0.668 | 0.695 | 0.747 | -0.160 | -0.178 | -0.347 | -0.120 | -0.276 | -0.268 |
| Methylotrophy | 0.162 | -0.401 | 0.095 | 0.496 | 0.990*** | 0.837* | -0.046 | -0.079 | 0.195 | -0.028 | 0.430 | 0.099 |
| Methanotrophy | 0.130 | -0.406 | 0.045 | 0.397 | 0.980*** | 0.760 | -0.057 | -0.097 | 0.216 | -0.043 | 0.451 | 0.101 |
| Fermentation | -0.084 | -0.335 | 0.189 | 0.891* | 0.636 | 0.855* | -0.075 | -0.077 | -0.387 | -0.028 | -0.369 | -0.248 |
| Hydrocarbon Degradation | 0.130 | -0.406 | 0.045 | 0.397 | 0.980*** | 0.760 | -0.057 | -0.097 | 0.216 | -0.043 | 0.451 | 0.101 |
| Aromatic compound degradation | 0.820* | -0.873* | -0.820* | 0.432 | 0.259 | 0.233 | 0.890* | 0.883* | 0.554 | 0.908* | 0.342 | 0.764 |
| Cellulolysis | 0.879* | -0.776 | -0.879* | 0.179 | -0.105 | -0.119 | 0.978** | 0.981*** | 0.660 | 0.978*** | 0.370 | 0.854* |
| Hydrogenotrophic methanogenesis | -0.416 | 0.136 | 0.809 | 0.607 | 0.241 | 0.561 | -0.501 | -0.488 | -0.514 | -0.496 | -0.459 | -0.584 |
| Methanogenesis by CO_2_ reduction | -0.414 | 0.154 | 0.805 | 0.624 | 0.220 | 0.564 | -0.494 | -0.478 | -0.538 | -0.486 | -0.488 | -0.589 |
| Nitrogen fixation | 0.470 | -0.708 | -0.197 | 0.625 | 0.945*** | 0.822 | 0.309 | 0.279 | 0.397 | 0.328 | 0.505 | 0.382 |
| Nitrate reduction | -0.468 | 0.627 | 0.182 | -0.926** | -0.400 | -0.694 | -0.470 | -0.484 | -0.041 | -0.509 | 0.101 | -0.263 |
| Nitrogen respiration | -0.569 | 0.705 | 0.296 | -0.879* | -0.381 | -0.635 | -0.577 | -0.588 | -0.155 | -0.611 | 0.010 | -0.378 |
| Nitrate respiration | -0.569 | 0.705 | 0.296 | -0.879* | -0.381 | -0.635 | -0.577 | -0.588 | -0.155 | -0.611 | 0.010 | -0.378 |
| Nitrite respiration | 0.921** | -0.699 | -0.798 | -0.122 | 0.215 | -0.075 | 0.832* | 0.811 | 0.982*** | 0.813 | 0.915* | 0.970*** |
| Ureolysis | 0.681 | -0.435 | -0.451 | -0.191 | 0.405 | 0.069 | 0.473 | 0.446 | 0.868* | 0.453 | 0.980*** | 0.737 |
| Chitinolysis | 0.971*** | -0.747 | -0.885* | -0.048 | 0.099 | -0.113 | 0.938** | 0.926** | 0.937** | 0.926** | 0.793 | 1.000*** |
| Aerobic ammonia oxidation | 0.512 | -0.308 | -0.514 | -0.599 | 0.056 | -0.393 | 0.409 | 0.376 | 0.861* | 0.363 | 0.900* | 0.671 |
| Nitrification | -0.081 | 0.275 | -0.119 | -0.843* | -0.038 | -0.486 | -0.170 | -0.200 | 0.363 | -0.206 | 0.555 | 0.129 |
| Aerobic nitrite oxidation | -0.236 | 0.413 | -0.003 | -0.845* | -0.061 | -0.474 | -0.314 | -0.342 | 0.200 | -0.345 | 0.419 | -0.028 |
| Chlorate reducers | -0.803 | 0.796 | 0.505 | -0.691 | -0.528 | -0.602 | -0.727 | -0.726 | -0.495 | -0.755 | -0.409 | -0.662 |
| Animal parasites or symbionts | 0.749 | -0.859* | -0.743 | 0.532 | 0.296 | 0.318 | 0.828* | 0.823 | 0.446 | 0.851* | 0.235 | 0.671 |
| Human Pathogens all | 0.749 | -0.859* | -0.743 | 0.532 | 0.296 | 0.318 | 0.828* | 0.823 | 0.446 | 0.851* | 0.235 | 0.671 |
| Predatory or exoparasitic | 0.972*** | -0.854* | -0.756 | 0.308 | 0.390 | 0.267 | 0.894* | 0.881* | 0.833* | 0.898* | 0.731 | 0.925** |

**Desu**: *Desulfomonile*; **Methy**: *Methylocystis*; **Hypho**: *Hyphomicrobium*; **Pheny**: *Phenylobacterium*; **Sphi**: *Sphingomonas*; **Thio**: *Thiobacillus*; **Aqua**: *Aquabacterium*; **monas**: *Thermomonas*; **Lyso**: *Lysobacter*; **gutta**: *Thermogutta*; **Gai**: *Gaiella*.

**Table S3-2** Pearson correlation between dominant class and functional groups

| **Functional group** | **Anaero** | **Alpha** | **Gamma** | **Delta** | **Planct** | **Beta** | **Actino** | **Gp16** | **Clostr** | **Gp6** | **Gp17** | **Bacilli** |
| --- | --- | --- | --- | --- | --- | --- | --- | --- | --- | --- | --- | --- |
| Chemoheterotrophy | -0.713 | 0.981  *** | 0.240 | -0.651 | -0.887  *** | 0.527 | 0.866  *** | 0.818  *** | 0.399 | -0.883  *** | -0.621 | 0.585 |
| Aerobic chemoheterotrophy | -0.868  *** | 0.785 | 0.648 | -0.367 | -0.684 | 0.864  *** | 0.954  *** | 0.988  *** | -0.094 | -0.789 | -0.863  *** | 0.074 |
| Respiration of sulfur compounds | 0.727 | -0.886  *** | -0.422 | 0.582 | 0.739 | -0.689 | -0.957  *** | -0.968  *** | -0.176 | 0.776 | 0.726 | -0.153 |
| Sulfate respiration | 0.737 | -0.867  *** | -0.449 | 0.564 | 0.720 | -0.712 | -0.964  *** | -0.977  *** | -0.135 | 0.773 | 0.751 | -0.121 |
| Thiosulfate respiration | 0.348 | -0.888  *** | 0.126 | 0.669 | 0.779 | -0.130 | -0.546 | -0.520 | -0.746 | 0.560 | 0.100 | -0.598 |
| Sulfur respiration | 0.348 | -0.888  *** | 0.126 | 0.669 | 0.779 | -0.130 | -0.546 | -0.520 | -0.746 | 0.560 | 0.100 | -0.598 |
| Sulfite respiration | -0.471 | 0.224 | 0.331 | -0.351 | 0.046 | 0.495 | 0.608 | 0.630 | -0.258 | -0.228 | -0.807  ** | -0.359 |
| Dark oxidation of Sulfur compounds | -0.873  *** | 0.577 | 0.898  *** | 0.060 | -0.623 | 0.981  *** | 0.781 | 0.869  *** | -0.399 | -0.695 | -0.739 | -0.079 |
| Dark sulfide oxidation | -0.866  *** | 0.562 | 0.905  *** | 0.076 | -0.613 | 0.982  *** | 0.772 | 0.862  *** | -0.413 | -0.684 | -0.732 | -0.096 |
| Chloroplasts | 0.064 | 0.372 | -0.573 | -0.510 | -0.361 | -0.422 | -0.020 | -0.181 | 0.706 | -0.298 | 0.124 | 0.946  *** |
| Methylotrophy | -0.354 | 0.638 | -0.088 | -0.248 | -0.663 | 0.042 | 0.174 | 0.162 | 0.602 | -0.401 | 0.095 | 0.822  *** |
| Methanotrophy | -0.406 | 0.581 | -0.022 | -0.154 | -0.631 | 0.084 | 0.142 | 0.130 | 0.499 | -0.406 | 0.045 | 0.845  *** |
| Fermentation | 0.204 | 0.484 | -0.698 | -0.737 | -0.433 | -0.495 | 0.093 | -0.084 | 0.878  *** | -0.335 | 0.189 | 0.848  *** |
| Hydrocarbon degradation | -0.406 | 0.581 | -0.022 | -0.154 | -0.631 | 0.084 | 0.142 | 0.130 | 0.499 | -0.406 | 0.045 | 0.845  *** |
| Aromatic compound degradation | -0.631 | 0.808  ** | 0.249 | -0.693 | -0.690 | 0.532 | 0.912  *** | 0.820  *** | 0.181 | -0.873  *** | -0.820  *** | 0.382 |
| Cellulolysis | -0.589 | 0.619 | 0.466 | -0.503 | -0.529 | 0.680 | 0.927  *** | 0.879  *** | -0.144 | -0.776 | -0.879  *** | -0.041 |
| Hydrogenotrophic methanogenesis | 0.741 | -0.031 | -0.601 | -0.173 | -0.124 | -0.640 | -0.339 | -0.416 | 0.553 | 0.136 | 0.809  ** | 0.146 |
| Methanogenesis by CO_2_ reduction | 0.755 | -0.029 | -0.635 | -0.213 | -0.103 | -0.663 | -0.335 | -0.414 | 0.581 | 0.154 | 0.805  ** | 0.137 |
| Nitrogen fixation | -0.509 | 0.871  *** | 0.035 | -0.476 | -0.876  *** | 0.247 | 0.517 | 0.470 | 0.583 | -0.708 | -0.197 | 0.819  *** |
| Nitrate reduction | 0.027 | -0.773 | 0.366 | 0.949  *** | 0.658 | 0.074 | -0.610 | -0.468 | -0.739 | 0.627 | 0.182 | -0.482 |
| Nitrogen respiration | 0.129 | -0.817  *** | 0.251 | 0.937  *** | 0.706 | -0.048 | -0.704 | -0.569 | -0.659 | 0.705 | 0.296 | -0.452 |
| Nitrate respiration | 0.129 | -0.817  *** | 0.251 | 0.937  *** | 0.706 | -0.048 | -0.704 | -0.569 | -0.659 | 0.705 | 0.296 | -0.452 |
| Nitrite respiration | -0.921  *** | 0.658 | 0.825  *** | -0.077 | -0.628 | 0.959  *** | 0.837  *** | 0.921  *** | -0.287 | -0.699 | -0.798 | -0.019 |
| Ureolysis | -0.823  *** | 0.539 | 0.742 | 0.147 | -0.537 | 0.804  ** | 0.537 | 0.681 | -0.188 | -0.435 | -0.451 | 0.024 |
| Chitinolysis | -0.875  *** | 0.678 | 0.759 | -0.226 | -0.610 | 0.931  *** | 0.920  *** | 0.971  *** | -0.263 | -0.747 | -0.885  *** | -0.052 |
| Aerobic ammonia oxidation | -0.767 | 0.153 | 0.960  *** | 0.539 | -0.265 | 0.887  *** | 0.362 | 0.512 | -0.672 | -0.308 | -0.514 | -0.239 |
| Nitrification | -0.445 | -0.345 | 0.625 | 0.824  *** | 0.267 | 0.433 | -0.252 | -0.081 | -0.656 | 0.275 | -0.119 | -0.231 |
| Aerobic nitrite oxidation | -0.323 | -0.454 | 0.485 | 0.839  *** | 0.392 | 0.276 | -0.400 | -0.236 | -0.600 | 0.413 | -0.003 | -0.211 |
| Chlorate reducers | 0.516 | -0.974  *** | -0.067 | 0.810  *** | 0.829  *** | -0.380 | -0.861  *** | -0.803  ** | -0.538 | 0.796 | 0.505 | -0.458 |
| Animal parasites or symbionts | -0.536 | 0.807  ** | 0.123 | -0.762 | -0.689 | 0.415 | 0.865  *** | 0.749 | 0.283 | -0.859  *** | -0.743 | 0.456 |
| Human Pathogens all | -0.536 | 0.807  ** | 0.123 | -0.762 | -0.689 | 0.415 | 0.865  *** | 0.749 | 0.283 | -0.859  *** | -0.743 | 0.456 |
| Predatory or exoparasitic | -0.808  ** | 0.905  *** | 0.514 | -0.495 | -0.812  *** | 0.762 | 0.963  *** | 0.972  *** | 0.113 | -0.854  *** | -0.756 | 0.237 |

**Anaero**: Anaerolineae, **Alpha**: Alphaproteobacteria, **Gamma**: Gammaproteobacteria, **Delta**: Deltaproteobacteria, **Planct**: Planctomycetia, **Beta**: Betaproteobacteria, **Actino**: Actinobacteria, **Gp16**: Acidobacteria_Gp16, **Clostr**: Clostridia, **Gp6**: Acidobacteria_Gp6, **Gp17**: Acidobacteria_Gp17, **Cyano**: Cyanobacteria, **Caldili**: Caldilineae, **Bacilli**: Bacilli.

Significant level: ***, p < 0.001; **, 0.001< p < 0.01; *, 0.01< p <0.05.

**Supplementary Figures**


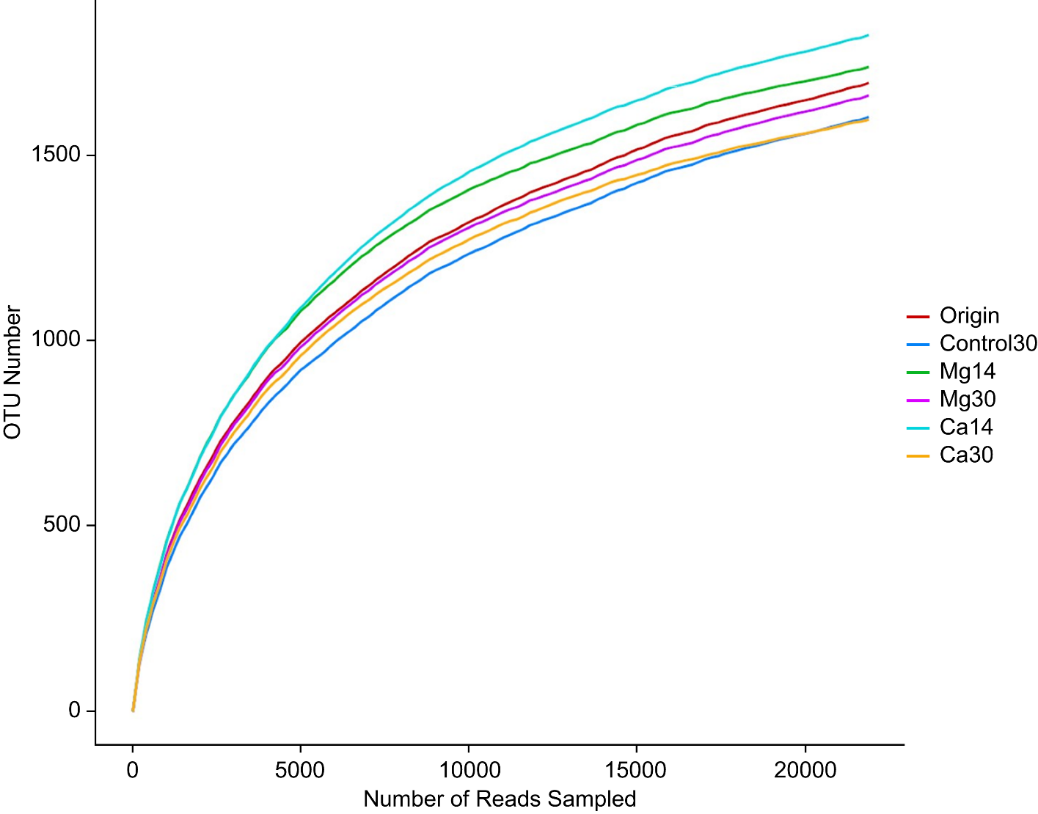


**Fig. S1** Rarefaction curve showing the number of observed OTUs among the four sedimental microcosms in this study. OTUs, operational taxonomic units.


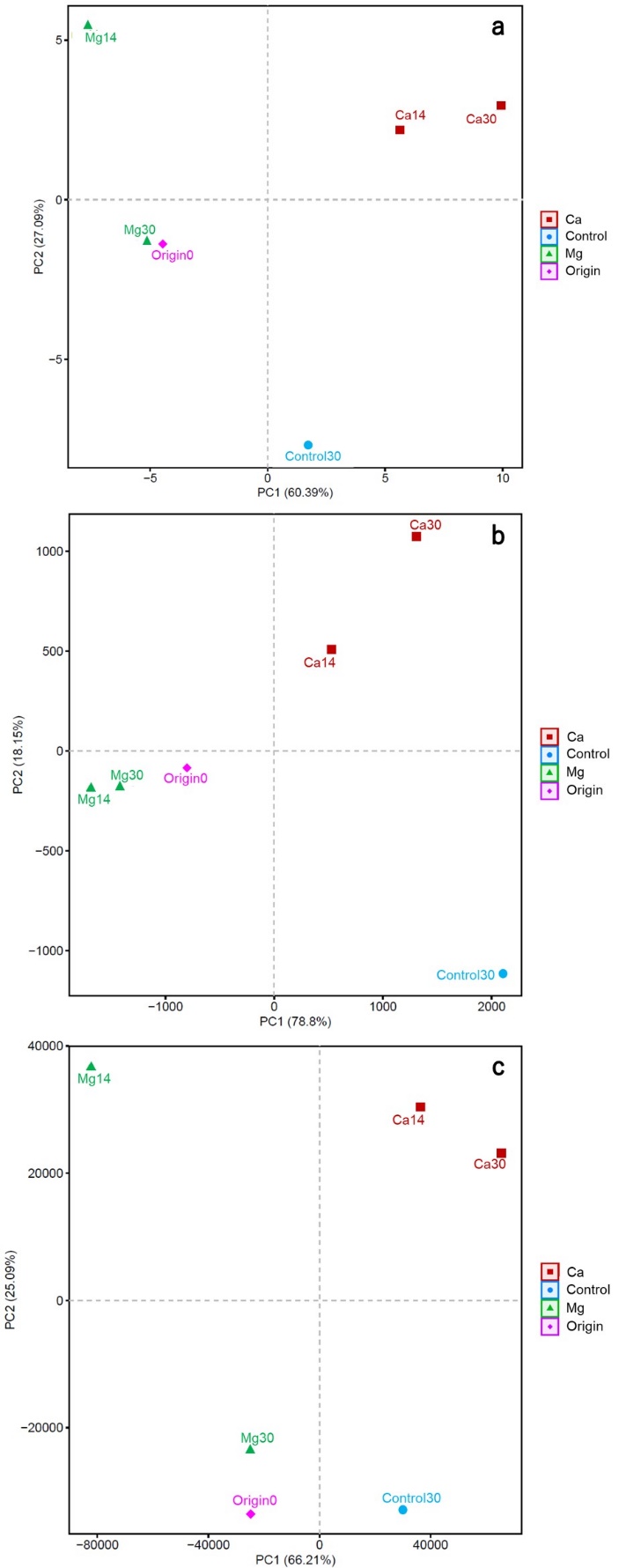


**Fig. S2** Principal component analysis (PCA) demonstrated the dissimilarity of the origin sediment and the control, Ca, and Mg microcosm samples for (a) microbial community composition at the genus level; (b) potential microbial ecological function based on FAPROTAX prediction; (c) potential microbial ecological function based on PICRUSt prediction. PCA analysis was based on Bray-Curtis dissimilarity.


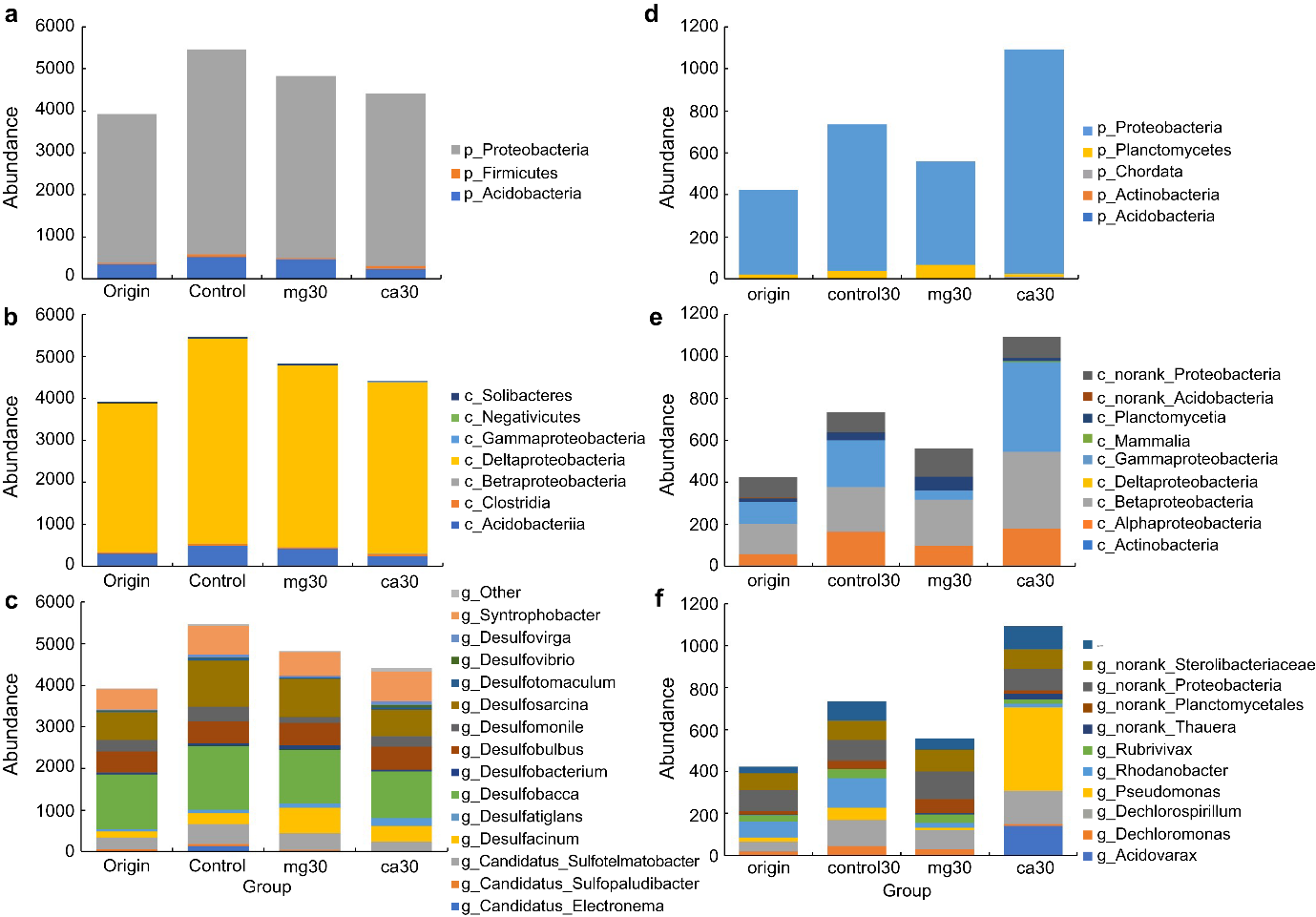


**Fig. S3** Composition of SRB (a–c) and NRB (d–f) at the phylum, class, and genus level according to *dsrB* and *nirS* functional gene sequencings SRB, sulfate-reducing bacteria; NRB, nitrate-reducing bacteria


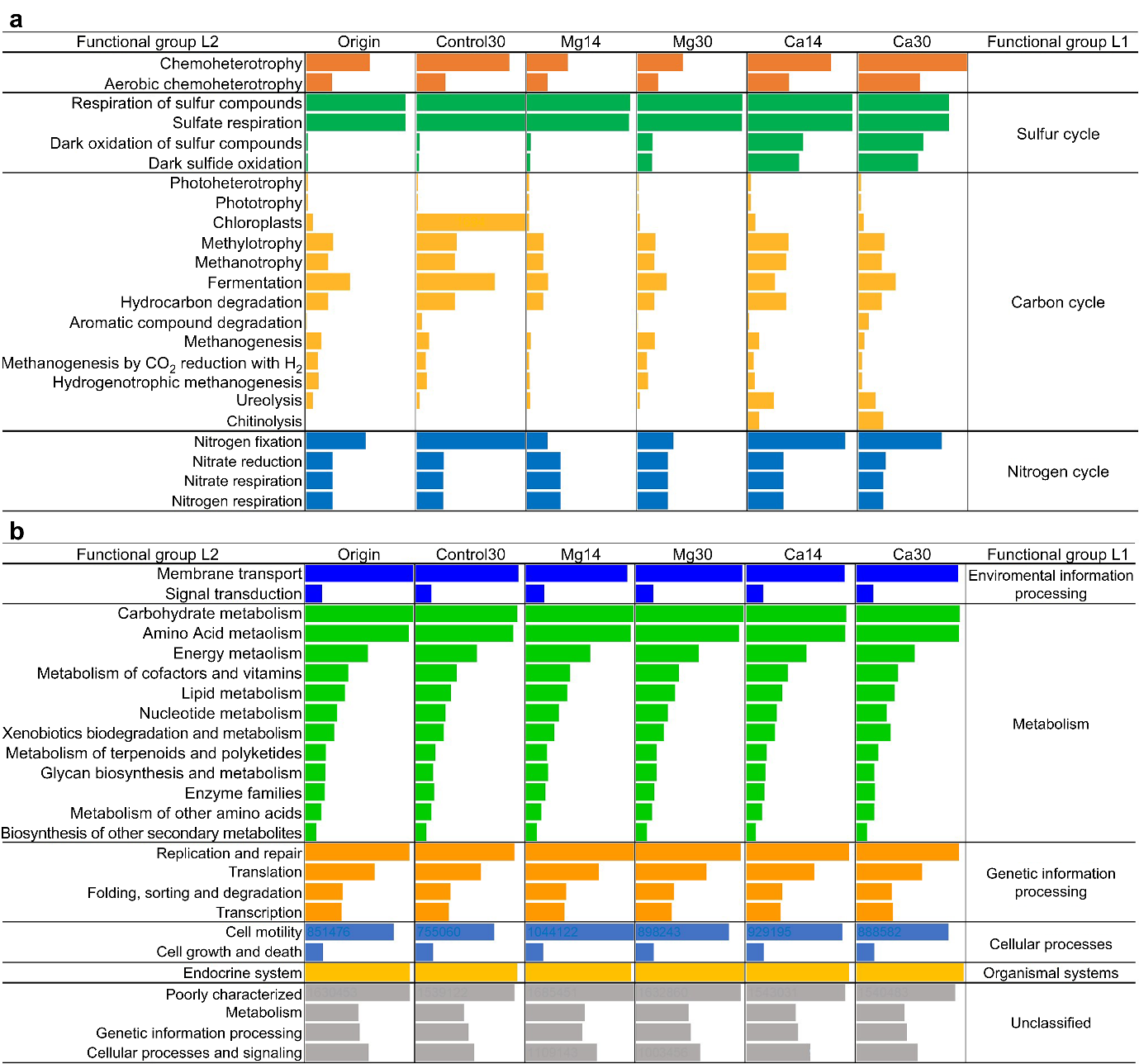


**Fig. S4** Composition of dominant bacterial functional groups in different samples shown by an abundance bar plot with clustering analysis based on FAPROTAX (a) and PICRUSt (b) functional predictions. Length of bar indicates the abundance of functional groups


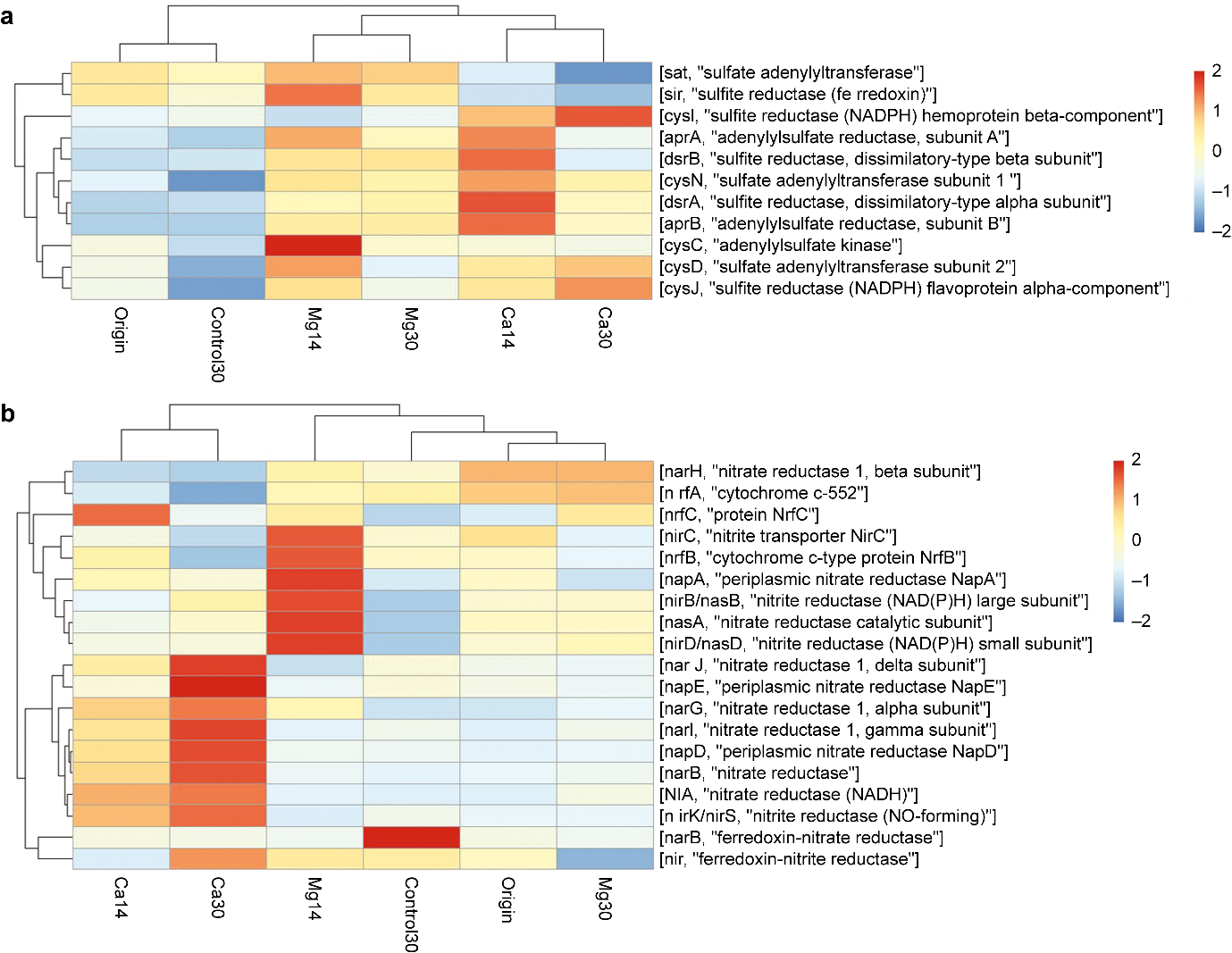


**Fig. S5** Genes potentially involved in the metabolism of sulfate reduction (a) and nitrate reduction (b) according to PICRUSt prediction


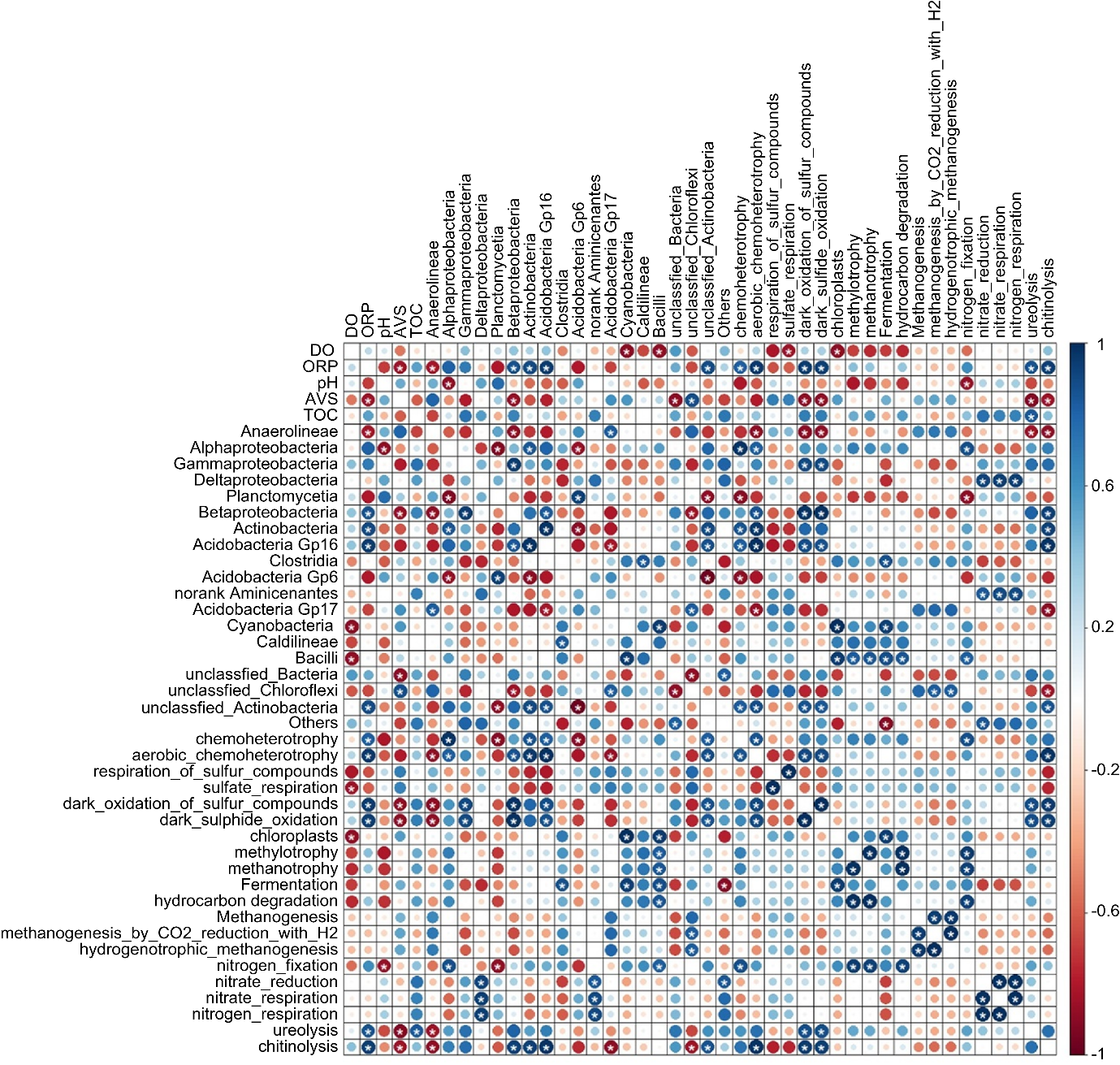


**Fig. S6** Pearson correlation analysis of dominant bacteria at the class level, dominant functional groups, and sediment environmental parameters. The size and color of each circle represents the absolute value of the correlation coefficient. Blue represents a positive correlation and red represents a negative correlation. White “*” symbol represents the results with a p-value < 0.05 and an absolute value of the correlation coefficient ≥ 0.80.


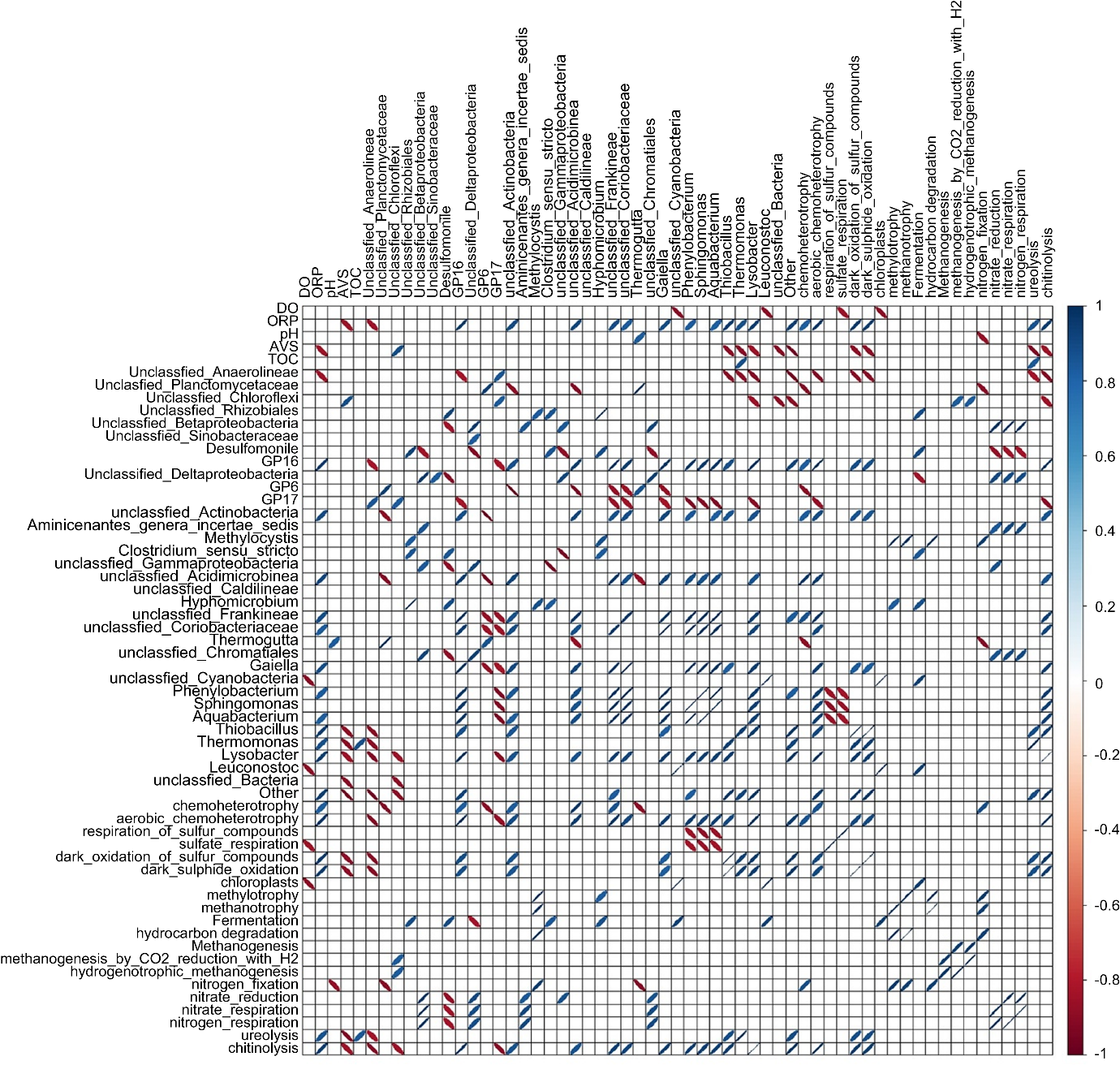


**Fig. S7** Pearson correlation analysis of dominant bacteria at the genus level, dominant functional groups, and sediment environmental parameters. The width of the ellipse represents the absolute value of the correlation coefficient; the larger the absolute value of the correlation, the narrower the ellipse. Blue ellipses skewed to the right represent a positive correlation, whereas red ellipses skewed to the left represent a negative correlation. Only those results with a p-value < 0.05 and an absolute value of the correlation coefficient ≥ 0.80 are shown.


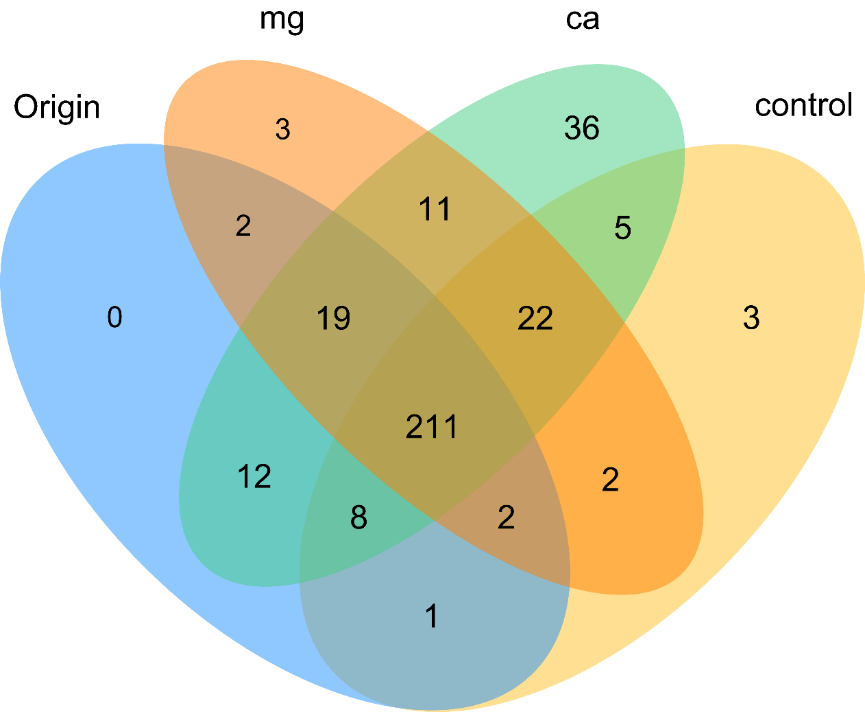


**Fig. S8** Venn diagram showing shared and unique genera at 97% identity among the four sedimental microcosms in this study

Origin: original sediment; mg: Mg(OH)_2_ treatment microcosms; ca: Ca(NO_3_)_2_ treatment microcosms; control: control treatment microcosms
